# The effects of spatially-constrained treatment regions upon a model of wombat mange

**DOI:** 10.1007/s00285-024-02078-9

**Published:** 2024-04-02

**Authors:** Ivy J. Hindle, Lawrence K. Forbes, Stephen J. Walters, Scott Carver

**Affiliations:** 1 Department of Life Sciences, University of Tasmania, Hobart, TAS 7001 Australia; 2 Department of Mathematics and Physics, University of Tasmania, Hobart, TAS 7001 Australia; 3 Odum School of Ecology, University of Georgia, Athens, GA 30602 USA

**Keywords:** Sarcoptic mange, Wombats, Spatial variation, Treatment, Mathematical model, Terrain knowledge, 92D30, 92D25, 65M70

## Abstract

The use of therapeutic agents is a critical option to manage wildlife disease, but their implementation is usually spatially constrained. We seek to expand knowledge around the effectiveness of management of environmentally-transmitted *Sarcoptes scabiei* on a host population, by studying the effect of a spatially constrained treatment regime on disease dynamics in the bare-nosed wombat *Vombatus ursinus*. A host population of wombats is modelled using a system of non-linear partial differential equations, a spatially-varying treatment regime is applied to this population and the dynamics are studied over a period of several years. Treatment could result in mite decrease within the treatment region, extending to a lesser degree outside, with significant increases in wombat population. However, the benefits of targeted treatment regions within an environment are shown to be dependent on conditions at the start (endemic vs. disease free), as well as on the locations of these special regions (centre of the wombat population or against a geographical boundary). This research demonstrates the importance of understanding the state of the environment and populations before treatment commences, the effects of re-treatment schedules within the treatment region, and the transient large-scale changes in mite numbers that can be brought about by sudden changes to the environment. It also demonstrates that, with good knowledge of the host-pathogen dynamics and the spatial terrain, it is possible to achieve substantial reduction in mite numbers within the target region, with increases in wombat numbers throughout the environment.

**Supplementary Information:**

The online version contains supplementary material available at 10.1007/s00285-024-02078-9.

## Introduction

The effective management of infectious pathogens in free-ranging animals has many complex challenges, such as the identification of diseased individuals or the removal or targeted treatment of diseased individuals (Wobeser 2002). An option for disease management is the use of therapeutic agents, such as anti-parasitic drugs (Martin et al. 2019; Skerratt 2003; Death et al. 2011); however, the delivery of therapeutic agents to free-ranging hosts is challenging in nature. Consequently, treatment programs in wildlife are often spatially restricted to confined areas of specified sub-populations (Martin et al. 2019). Understanding how effective spatially restricted treatment regimes actually are, for host populations, is therefore valuable for *in situ* disease management practice.

The parasitic mite *Sarcoptes scabiei*, causing the disease sarcoptic mange, is one of the most host-generalist of mammalian parasites (Astorga et al. 2018). This parasite has been documented to infect approximately one hundred and fifty mammalian species across the globe (Pence and Ueckermann 2002), and continues to have an expanding host species range (Tompkins et al. 2015; Martin et al. 2018b). The mite burrows into the host’s skin, leading to a range of pathological outcomes, including pruritus, alopecia, secondary bacterial infections and emaciation (Arlian and Morgan 2017; Astorga et al. 2018). It is both an animal welfare issue and occasional conservation issue, causing epizootics and host population declines (Pence and Ueckermann 2002). Known as scabies when infecting a human host, it is also among the thirty most prevalent human diseases (estimated point prevalence two hundred million people) (World Health Organisation 2020) and is considered a Neglected Tropical Disease by the World Health Organisation (Karimkhani et al. 2017).

Sarcoptic mange is the most important disease impacting the bare-nosed wombat (*Vombatus ursinus*) (Martin et al. 2018a), with an estimated mortality rate of 100% for untreated individuals (Martin et al. 2018a; Skerratt 2003). The parasite is believed to have been introduced to Australia by European colonists and their domestic animals (Skerratt et al. 1998; Fraser et al. 2016). Wombat populations that are exposed to mange experience a variety of population outcomes, including both epizootics with population decline and endemic disease with relatively stable populations (Driessen et al. 2021; Skerratt et al. 1998; Martin et al. 2018a). Transmission of *S. scabiei* is understood to be environmental via burrows (Skerratt et al. 1998), between which solitary wombats move every 4–10 days, sharing asynchronously. In situ attempts at disease management in wombats is relatively common at the individual level (Skerratt 2003), and there is increasing interest about pathogen management at population scales. For example, a recent population scale treatment attempt during an epizootic showed short-term pathogen management was achievable (Martin et al. 2019). In most instances, population scale *S. scabiei* management attempts in wombats will be spatially restricted, so understanding how this restriction impacts spatial disease dynamics can inform management interventions.

In this study, we seek to characterize the effects of a realistic treatment regime on a population of bare-nosed wombats suffering from endemic *S. scabiei* infection. This treatment will be spatially confined to a sub-region of the host range. We aim to expand upon the spatio-temporally varying model proposed by Hindle et al. (2022), by introducing treatment for disease and exploring the long-term dynamics introduced by a short-term, spatially restricted treatment plan. A common treatment regime for wombats focusses on the repeated delivery of treatment [(e.g. at least 1 ml of moxidectin per 10 kg of animal weight (Martin et al. 2019)] over a defined period (e.g. weekly for 12 weeks), which may be targeted at infected individuals or indiscriminately at a population scale. This treatment is applied topically to the wombat, usually via a burrow flap (Martin et al. 2019). We focus on indiscriminate treatment at a population scale [consistent with Martin et al. (2019)]. Two differing sub-regions are considered, one situated at the centre of the host population range, and the other at one corner, with the intention of discovering if one or the other results in a sustained reduction in mite population density and an improved outcome for the wombats.

The wombat–mite model and the governing partial differential equations that describe it are introduced in Sect. 2. The steady-state populations possible in this model, for uniform treatment, are briefly reviewed in Sect. 3 and follow the developments in Beeton et al. (2019). It is found that up to four steady states are possible, although one of these is non-physical since it would involve negative populations; the remaining three consist of a total-extinction state, a mite-free state and an endemic state in which infected wombats and mites co-exist. Driving the system toward the mite-free state is an obvious intention of this study. We briefly consider a linearization of the governing equations in Sect. 4, because the governing equations in Sect. 2 are nonlinear and so can only be solved approximately with the aid of computers. This linearized solution therefore provides an important check on the accuracy of the computationally obtained nonlinear results. Nevertheless, the linearized solution has severe restrictions, since it is only valid when populations remain close to their assumed steady-state values, and so numerical solutions are needed for the fully nonlinear model outlined in Sect. 2, and our method for generating such solutions is discussed in Sect. 5. Results of this work are presented in Sect. 6; we first compare the linearized solution with the nonlinear computational results, and then investigate the effects of treatment regimes. Some final remarks in Sect. 7 conclude the paper.

## The wombat–mite model

**Fig. 1:**
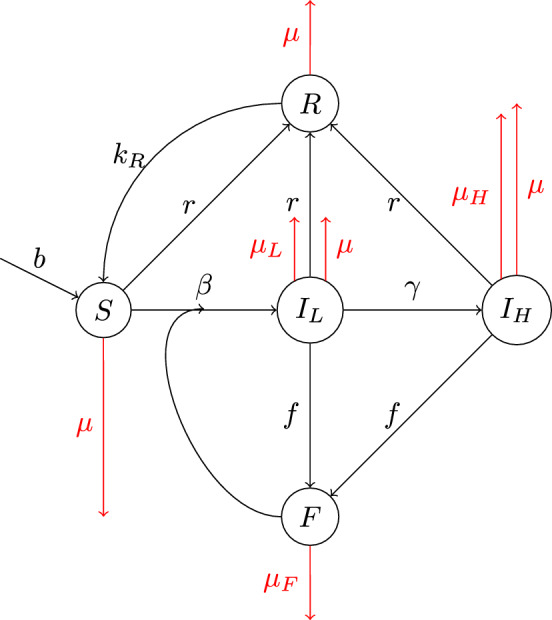
A modified version of the model developed in Hindle et al. (2022). Here the transmission method is environmental (indirect) and the population of the mites is modelled explicitly, to represent this indirect transmission. In this diagram, *S* represents the sub-population of susceptible wombats, $I_L$ are asymptomatic carriers of the disease, $I_H$ are symptomatic infected wombats, *F* represents the proportion of fomites existing in the environment, and *R* are the treated wombats. The quantity *r* is the treatment rate, $k_R$ is the relapse rate at which treated wombats re-enter the susceptible group, *b* is the birth rate of wombats, $\mu$ is the death rate without infection of wombats, $\mu _L$ is the death rate due to asymptomatic infection, $\mu _H$ is the death rate due to symptomatic infection, $\beta$ is the rate at which susceptible wombats are infected, $\gamma$ is the rate at which asymptomatic wombats become symptomatic, *f* is the rate that infected wombats drop fomites into the environment and $\mu _F$ is the rate that fomites within the environment die

A conceptual model illustrating the wombat–mite system and its dynamics is illustrated in Fig. 1, with rate parameters for the system described in Table 1. These parameters will be assumed to be those used for the results in Sect. 6, unless indicated in the text.

**Table 1:** Rate parameters for the PDE (see Eqs. (1)–(5) and Fig. 1) describing transmission of *S. scabiei* among wombats

Parameter	Symbol	Values	Source
Wombat spatial diffusion rate	$\sigma$	1/(1.5 $\times$ 365 $\times$ 0.5 $\times$ 0.5)	Banks et al. (2002)
Wombat birth rate	*b*	1/(3 $\times$ 365)	Beeton et al. (2019), Martin et al. (2019) and Triggs (2009)
Non-linear disease spread rate	$\beta$	0.01	Tamura et al. (2021)
Wombat death rate	$\mu$	1/(15 $\times$ 365)	Triggs (2009)
Disease progression rate	$\gamma$	1/30	Skerratt (2003) and Martin et al. (2019)
Exposed wombat death rate	$\mu _L$	1/60	Martin et al. (2019)
Infected wombat death rate	$\mu _H$	1/60	Martin et al. (2019)
Mite drop rate	*f*	1/5	Beeton et al. (2019)
Mite death rate	$\mu _F$	1/19	Arlian et al. (1984) and Browne et al. (2021)
Treatment rate	*r*	0.9/7	Martin et al. (2019)
Treatment relapse rate	$k_R$	1/7	Beeton et al. (2019)

All rates are in days, and the wombat spatial diffusion rate is in km/day

We create a system of nonlinear equations in two spatial dimensions and time, expanding upon the work of Hindle et al. (2022). The governing partial differential equations (PDEs) describing this system are:

1 $$\begin{aligned}{} & {} \frac{\partial S}{\partial t} = \sigma \nabla ^2 S + bN(1-N) - \frac{\beta SF}{1+F} - (\mu +r) S + k_R R \end{aligned}$$

2 $$\begin{aligned}{} & {} \frac{\partial I_L}{\partial t} = \sigma \nabla ^2 I_L + \frac{\beta SF}{1+F} - (\mu +\gamma +\mu _{L}+r )I_L \end{aligned}$$

3 $$\begin{aligned}{} & {} \frac{\partial I_H}{\partial t} = \sigma \nabla ^2 I_H + \gamma I_L - (\mu +\mu _H +r)I_H \end{aligned}$$

4 $$\begin{aligned}{} & {} \frac{\partial R}{\partial t} = \sigma \nabla ^2 R + \left( S+I_L+I_H \right) r - (\mu +k_R) R \end{aligned}$$

5 $$\begin{aligned}{} & {} \frac{\partial F}{\partial t} = \left( I_L+I_H \right) f - \mu _F F. \end{aligned}$$

Here, the quantities *r* and *f* are rate constants, as indicated in Fig. 1. The $\nabla ^2$ operator used in the above equations is the two dimensional Laplacian operator

$$\begin{aligned} \nabla ^2 \equiv \frac{\partial ^2}{\partial x^2} + \frac{\partial ^2}{\partial y^2} \end{aligned}$$

and represents the movement of wombats throughout the system. This movement is thus modelled as a diffusion process, with wombats spreading throughout the region as time progresses, governed by the spreading parameter $\sigma$ in Table 1.

The governing equations (1)–(5) describe the movement of the wombat populations within the spatial region as well as their change over time. The wombat population is split into four sub-populations, following conventional SEIR models of disease spread (Kermack and McKendrick 1927). Thus the total wombat population is *N*, and we postulate

6 $$\begin{aligned} N = S + I_L + I_H + R, \end{aligned}$$

in which *S* denotes the sub-population of wombats that are susceptible to, but not yet infected by *S. scabiei*. In addition, $I_L$ represents the wombats that are infected by the disease, but are carrying a sufficiently low population of mites that they would not be seen as being infected in a standard field study; in an SEIR model, our sub-population $I_L$ would thus correspond to the “exposed” group. The sub-population $I_H$ represents those wombats carrying a high mite load and so show symptoms of the disease, and *R* is the “recovered” group that have been treated and are therefore temporarily immune from immediate further infection by *S. scabiei*. Finally, the population index of the fomites is labelled *F* and represents the mites existing on a host body, surviving within the bedding chamber of wombat burrows (Martin et al. 2018a; Beeton et al. 2019). This population is modelled as a proxy for indirect environmental transmission of sarcoptic mange in wombat populations.

The transmission terms in Eqs. (1), (2) at which the susceptible wombats enter the lightly-infected class are modelled with the frequency-dependent rate $\beta F / (1+F)$. This is a self-limiting infection term, such that $F / (1+F) \rightarrow 1$ as $F \rightarrow \infty$, and is consistent with current understanding of mange transmission among wombats (Beeton et al. 2019; Martin et al. 2019; Tamura et al. 2021). The function is intended to reflect the environmental–transmission nature of *S. scabiei* in wombat populations, where an individual’s probability of exposure is related more to contact with contaminated burrow environments, rather than a direct encounter with another infected animal. Consequently, exposure occurs largely independently of wombat population density.

To approximate the physical conditions within which the wombats live, we consider a barrier around our spatial region of interest. For simplicity, this will be assumed to be a rectangular domain $-L< x < L$, $-B< y < B$, and the borders of this region will be supposed to prevent any wombat movement through them, although movement along the barrier will be allowed. These physical conditions that are being modelled can be considered to be a reserve that has been fenced in, or an area with natural barriers surrounding it, such as oceans, rivers, hills or dense forest. This reflects the actual situation for the wombats living at Narawntapu National Park (NNP), as detailed by Martin et al. (2018a). On the idealized rectangular boundaries considered in the present model, these restrictions are represented mathematically by the boundary conditions

7 $$\begin{aligned} \frac{\partial S}{\partial x} = \frac{\partial I_L}{\partial x} = \frac{\partial I_H}{\partial x} = \frac{\partial R}{\partial x} = 0 \quad \textrm{on} \quad x = \pm L \end{aligned}$$

and

8 $$\begin{aligned} \frac{\partial S}{\partial y} = \frac{\partial I_L}{\partial y} = \frac{\partial I_H}{\partial y} = \frac{\partial R}{\partial y} = 0 \quad \textrm{on} \quad y = \pm B. \end{aligned}$$

In order to complete the mathematical model of this situation, it is necessary to impose appropriate initial conditions. In this paper, we will consider conditions under which the system starts at one of its possible steady-state configurations (see Sect. 3), but mites are added into some portion of the physical-space domain. Furthermore, we wish to allow the possibility that our treatment rate may be a function both of space and time, so that $r \equiv r(x,y,t)$. This must be taken into account in the numerical solution technique (see Sect. 5), and physically represents the fact that treatment may only be offered over some fraction of the total region available to the wombats, and that the treatment is only able to be provided over short times. This reflects the actual treatment regime attempted for the wombats living in Narawntapu National Park, documented in Martin et al. (2019), in which treatment stations (burrow flaps) were refilled weekly; this treatment ran for 12 weeks in total, and the therapeutic agent (moxidectin) was observed to drop exponentially in efficacy as each week passed, since the insecticide was degraded by ultraviolet light. We discuss the results obtained with this present model, for short bursts of treatment applied periodically to the system, in Sect. 6.2.

Some comments are required, too, concerning the reasoning behind the parameter values assumed in this current model, as outlined in Table 1. Firstly, the wombat spatial diffusion rate $\sigma$ is largely due to females relocating after successfully rearing a joey, and they move approximately half a kilometre away (Banks et al. 2002). The wombat birth rate *b* is one joey per female per 1.5 years, and the death rate $\mu$ is based on a wombat lifespan of 15 years. The transmission rate $\beta$ is consistent with that assumed by Beeton et al. (2019), and the disease progression rate $\gamma$ is based on a 30 day window before symptoms become clinically visible ($I_L$ moves to $I_H$). The death rate $\mu _H$ associated with mange is 60 days when showing clinical signs, and thus infection to mortality is about 90 days. The mite drop rate *f* is consistent with Beeton et al. (2019). To estimate a constant value for the treatment rate *r*, it was assumed that within the treatment region, 90% of the theoretical maximum treatment was delivered each 7 days (Martin et al. 2019). The treatment relapse rate $k_R$ is based on the treatment on a wombat lasting 7 days before the animal could return to the susceptible class and potentially be re-infected by *S. scabiei* (Beeton et al. 2019; Martin et al. 2019), and the environmental mite death rate $\mu _F$ is for optimal environmental conditions, amounting to 19 days based on laboratory studies (Arlian et al. 1984; Browne et al. 2021).

## The steady-state populations

The governing system of equations (1)–(5) in Sect. 2 allows several equilibrium population states to exist, in situations where the treatment rate *r* is constant across the entire solution domain. These were discussed briefly by Beeton et al. (2019). For completeness, and in view of their importance in the present model, they will be discussed briefly in this section.

Steady-state populations occur when the sub-populations *S*, $I_L$, $I_H$, *R* and the mite density *F* are independent of spatial variables *x* and *y* and time *t*. The treatment rate *r*, too, must be assumed to be constant. Then all the derivatives in (1)–(5) are set to zero, leaving a system of five nonlinear algebraic equations for the equilibrium populations $\left( S_{eq}, I_{L,eq}, I_{H,eq}, R_{eq}, F_{eq} \right)$.

It then follows at once from (3) that

9 $$\begin{aligned} I_{L,eq} = \frac{\theta _H}{\gamma } I_{H,eq} \end{aligned}$$

and (5) similarly yields

10 $$\begin{aligned} F_{eq} = \frac{f}{\mu _F} \left( \frac{\theta _H + \gamma }{\gamma } \right) I_{H,eq} \equiv \zeta _H I_{H,eq}. \end{aligned}$$

In these two expressions, and elsewhere in this paper, it is convenient to define the intermediate constants

11 $$\begin{aligned} \theta _L= & {} \mu + \mu _L + r \nonumber \\ \theta _H= & {} \mu + \mu _H + r. \end{aligned}$$

We have also created the constant

12 $$\begin{aligned} \zeta _H = \frac{f \left( \theta _H + \gamma \right) }{\mu _F \gamma }. \end{aligned}$$

The steady-state form of Eq. (2) is simply

$$\begin{aligned} \beta S_{eq} F_{eq} = \left( \theta _L + \gamma \right) \left( 1 + F_{eq} \right) I_{L,eq} \end{aligned}$$

and when (9), (10) are used in this expression, we obtain

$$\begin{aligned} \beta \gamma \zeta _H S_{eq} I_{H,eq} = \theta _H \left( \theta _L + \gamma \right) \left( 1 + \zeta _H I_{H,eq} \right) I_{H,eq}. \end{aligned}$$

This equation presents two possible solutions; either

13 $$\begin{aligned} I_{H,eq} = 0 \end{aligned}$$

or else

14 $$\begin{aligned} S_{eq} = \frac{\theta _H \left( \theta _L + \gamma \right) }{\beta \gamma } \left( \frac{1}{\zeta _H} + I_{H,eq} \right) . \end{aligned}$$

These two options are now studied using the remaining steady-state equations.

The simple choice in (13) is substituted into the steady-state forms of Eqs. (1) and (4), where they are easily seen to reveal two equilibrium solutions. The first of these is the total extinction case

15 $$\begin{aligned} \left( S_{eq}, I_{L,eq}, I_{H,eq}, R_{eq}, F_{eq} \right) = (0, 0, 0, 0, 0 ) \end{aligned}$$

with total wombat population $N_{eq} = 0$. The second equilibrium solution from this choice is seen to be

16 $$\begin{aligned}{} & {} I_{L,eq} = I_{H,eq} = F = 0; \nonumber \\{} & {} S_{eq} = \frac{b - \mu }{b K}; \quad R_{eq} = \frac{r(b - \mu )}{\left( \mu + k_R \right) b K}; \quad N_{eq} = \frac{b - \mu }{b}. \end{aligned}$$

Here, we have defined a further intermediate constant

17 $$\begin{aligned} K = \frac{\mu + k_R + r}{\mu + k_R} \end{aligned}$$

to simplify the notation. This second equilibrium (16) is surely the steady-state outcome to be desired, since it represents a situation in which the wombats live their lives free from mites.

The other choice (14) can give rise to circumstances in which both wombats and mites co-exist. It therefore needs to be explored, although the algebra is a little tedious. The steady-state (derivative-free) forms of the model equations (2), (3), (5) give

18 $$\begin{aligned}{} & {} S_{eq} = \mathcal {M} \left( 1 + \zeta _H I_{H,eq} \right) ; \quad { I_{L,eq} = \frac{\theta _H}{\gamma } I_{H,eq}; \quad } \nonumber \\{} & {} F_{eq} = \zeta _H I_{H,eq} {; \quad R_{eq} = \frac{r}{\mu + k_R} \left( \mathcal {M} + \mathcal {Z} I_{H,eq} \right) } \end{aligned}$$

and it follows from (6) that the total wombat number can be expressed as

19 $$\begin{aligned} N = K \left( \mathcal {M} + \mathcal {Z} I_{H,eq} \right) . \end{aligned}$$

In Eqs. (18) and (19) we have defined further intermediate constants

20 $$\begin{aligned} \mathcal {M} = \frac{\theta _H \left( \theta _L + \gamma \right) }{\beta \gamma \zeta _H}; \quad { \mathcal {Z} = \frac{\beta \left( \theta _H + \gamma \right) + \theta _H \left( \theta _L + \gamma \right) }{\beta \gamma } } \end{aligned}$$

and also made use of the constants defined in (11) and (12). These expressions (18) and (19) are substituted into the steady-state form of (1), and it is convenient to define a new quantity

21 $$\begin{aligned} \xi = \mathcal {M} + \mathcal {Z} I_{H,eq} \end{aligned}$$

which may be shown to satisfy the equation

22 $$\begin{aligned} b K^2 \xi ^2 + \mathbb {T} \xi - \frac{\mathcal {M}}{\gamma \mathcal {Z}} \left( \theta _H \mu _L + \gamma \mu _H \right) = 0. \end{aligned}$$

Here, we have defined

23 $$\begin{aligned} \mathbb {T} = -b K - \frac{k_R r}{\mu + k_R} + \frac{\zeta _H \mathcal {M}}{\mathcal {Z}} \left( \beta + \mu + r \right) \end{aligned}$$

and the constant *K* is as given in (17).

Since (22) is simply a quadratic equation, its two solutions can be written at once in the form

24 $$\begin{aligned} {\xi = \frac{1}{2b K^2} \left[ - \mathbb {T} \pm \sqrt{ \mathbb {T}^2 + 4b K^2 \frac{\mathcal {M}}{\gamma \mathcal {Z}} \left( \theta _H \mu _L + \gamma \mu _H \right) } \right] . } \end{aligned}$$

It is clear that both the solutions in (24) are real, and that the plus sign results in a positive value for $\xi$ and the negative sign always gives $\xi$ negative. Once $\xi$ has thus been determined, the equilibrium population $I_{H,eq}$ is recovered from the expression (21), after which all the other populations can be obtained from (18) and (19).

The minus sign option in the solution (24) always gives $\xi < 0$ and therefore always results in negative values for the sub-population $I_{H,eq}$ in (21). Consequently, this solution has no biological significance and can be discarded. However, the other solution, obtained from the choice of the plus sign in (24), is able to give positive and biologically relevant values for all the populations in this model, for appropriate choices of the parameters. This shows that a steady state is indeed possible, in which mites are endemic in the system. The aim of our treatment regime in such circumstances is to effect a movement from this endemic steady state to the mite-free state given in (16).

**Fig. 2:**
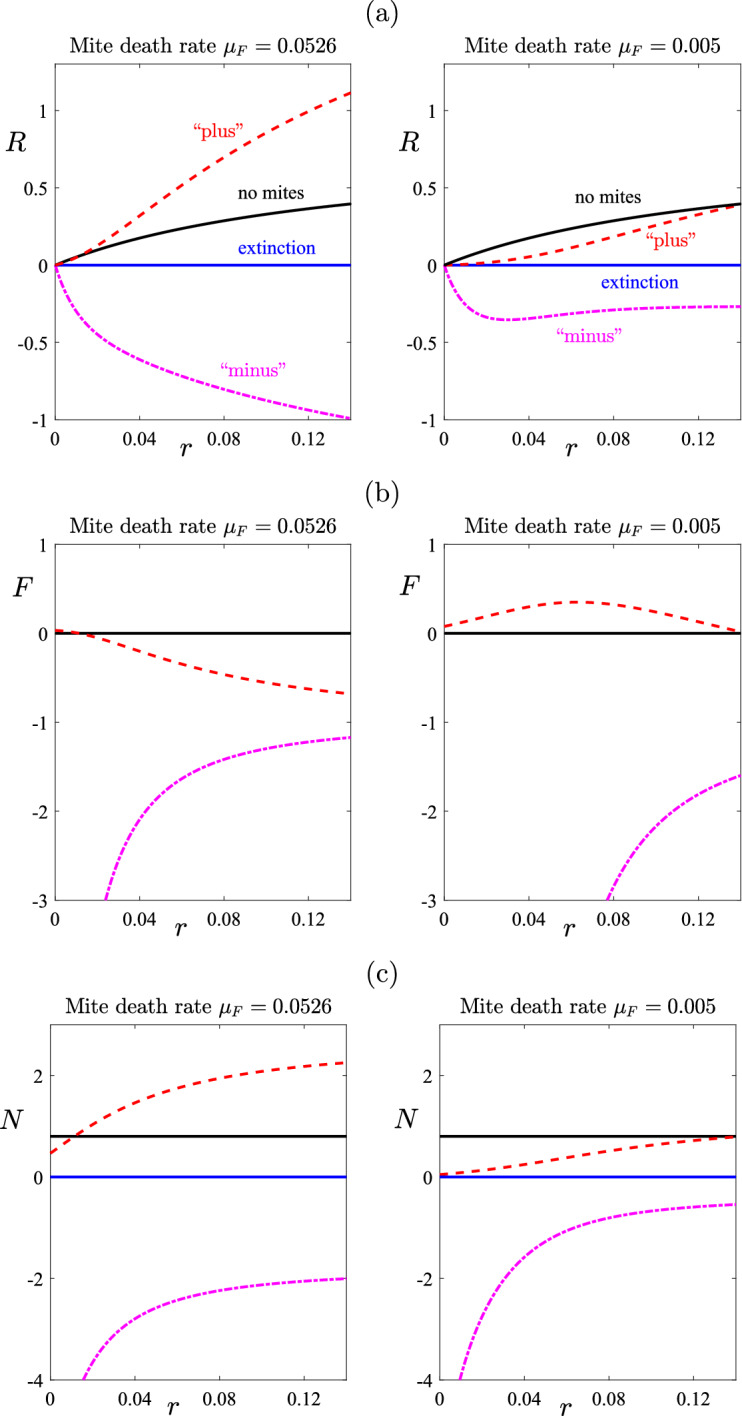
Equilibrium steady-state: **a** recovered sub-population *R*, **b** fomite population *F* and **c** total wombat population *N*, obtained using the parameters in Table 1. Results are shown for the mite death rate $\mu _F = 1/19 \approx 0.0526$ given in the Table, and also for the smaller value $\mu _F = 0.005$ discussed in the text

Figure 2 shows how some steady-state populations are affected by the treatment rate *r* (here assumed constant over the entire domain). We show the recovered sub-population *R* of wombats, the population density *F* of mites and the total wombat population *N* in parts (a), (b) and (c) respectively. On the left of each figure, the mite death rate has been set to the value $\mu _F = 1/19 \approx 0.0526$ given in Table 1, and the diagrams on the right have used the smaller value $\mu _F = 0.005$ as a comparison. The solid line (blue online) represents the total extinction equilibrium (15) and the other (black) solid line corresponds to the mite-free equilibrium (16). The two dashed lines come from the solution (24), and these represent the two endemic states. The formula with the minus sign in (24) gives negative mite and wombat populations, as discussed above, and these have no biological meaning, although for completeness we have nevertheless drawn those unphysical negative populations in Fig. 2. We observe, too, that for the steady-state populations obtained with $\mu _F = 1/19 \approx 0.0526$ on the left-hand side, the positive sign in the solution (24) gives an endemic equilibrium in which the recovered population *R* and total population *N* are both above the carrying capacity $N=1$, when the treatment rate *r* is sufficiently large; however, such an unphysical situation would not occur, since in those cases the mite population *F* has fallen below zero. Instead, the solution would revert to the mite-free equilibrium (16) for these larger treatment rates, exactly as desired. In addition, it is interesting to note that this “plus” endemic equilibrium, under our parameter values and for the case $\mu _F = 1/19 \approx 0.0526$, is highly sensitive (unstable) to the introduction of even small amounts of treatment.

The smaller mite death-rate $\mu _F = 0.005$ on the right-hand side of each diagram in Fig. 2 is almost certainly unphysical, since it would represent free mites living in the environment for 200 days, and this has not been encountered in our observations. In spite of the fact that it is therefore not of biological interest, it has nevertheless been included here so as to illustrate the mathematical dynamics possible in a model such as this. When $\mu _F = 0.005$ with treatment rates in about the interval $0< r < 0.1$, it is clear that one of three possible steady states could occur. The populations could fall to extinction (15), and the mite-free population (16) is at least a mathematically possible outcome; the stable state, however, is the endemic one indicated by the (red) dashed line, in which mites and wombats co-exist in this interval of treatment rates *r*. This corresponds to the “plus” sign in (24), which the linearized solution of the next Sect. 4 shows to be the stable one for $0< r < 0.1$. It is interesting to observe, too, that the mite population *F* actually first *increases* with increasing treatment rate *r*, as shown with the (red) dashed line in part (b) for mite death rate $\mu =0.005$. This somewhat paradoxical situation comes about because, with low treatment rates *r*, the number of wombats increases slightly, making more hosts available for mite infestation. In particular, the numbers $I_L$ and $I_H$ of exposed and infected wombats increase, and it follows from (5) that the growth rate of mites increases even as the wombat numbers start to recover. Eventually, however, as the treatment rate *r* is increased still further, the mite numbers do fall away below zero, at which point the mite-free equilibrium (16) becomes the state that is achieved.

## A linearized approximation

In this section, we develop an approximation to the full system of Eqs. (1)–(5) presented in Sect. 2, based on an assumption that the populations never stray far from (one of) their equilibrium values calculated in Sect. 3. This will give rise to a system of linear partial differential equations, which will be solved essentially exactly, although computer evaluation of the final solution will still be required. The reason for creating and solving this approximate model is primarily to serve as an important check on the accuracy of the numerical solutions to the full nonlinear model (1)–(5) that are needed later in the paper. Thus the linearized solution of this section is a necessary component in the proper analysis of our nonlinear model; nevertheless, it is quite limited in its scope, because it is only able to assess the behaviour of the system close to one of its equilibria.

It will be supposed here that a steady-state situation has been achieved, in which the populations of the fomites, as well as each of the sub-populations of the wombats, has reached equilibrium, as in Sect. 3. This equilibrium state is then disturbed, and here, it will be assumed that the perturbation remains small in comparison to the equilibrium population sizes. The size of this disturbance is estimated by some dimensionless parameter $\varepsilon$ that measures the change in a population from its equilibrium value. Each population *S*(*x*, *y*, *t*) and so on is then represented mathematically as a small perturbation to its equilibrium level, so that, overall

25 $$\begin{aligned} S(x,y,t)= & {} S_{eq} + \varepsilon S_1 (x,y,t) + \mathcal {O}(\varepsilon ^2) \nonumber \\ I_L (x,y,t)= & {} I_{L,eq} + \varepsilon I_{L1} (x,y,t) + \mathcal {O}(\varepsilon ^2) \nonumber \\ I_H (x,y,t)= & {} I_{H,eq} + \varepsilon I_{H1} (x,y,t) + \mathcal {O}(\varepsilon ^2) \nonumber \\ R(x,y,t)= & {} R_{eq} + \varepsilon R_1 (x,y,t) + \mathcal {O}(\varepsilon ^2) \nonumber \\ F(x,y,t)= & {} F_{eq} + \varepsilon F_1 (x,y,t) + \mathcal {O}(\varepsilon ^2). \end{aligned}$$

The task is now to find each of the perturbation functions $S_1 (x,y,t)$ and so on in this expansion. If $S_1$ grows larger as time increases, then the solution *S* moves further away from its equilibrium population $S_{eq}$ and as a result, that equilibrium is unstable. If, however, $S_1$ decreases with time, then the solution *S* approaches its equilibrium value $S_{eq}$ which consequently is stable.

These perturbed expressions in the representation (25) are substituted into the fully nonlinear system (1)–(5) and only terms of the first power in $\varepsilon$ are retained. This process is described by Hindle et al. (2022, Appendix A) and, after some algebra, yields

26 $$\begin{aligned} \frac{\partial S_1}{\partial t}= & {} \sigma \nabla ^2 S_1 + b N_1 \left( 1 - 2 N_{eq} \right) - \frac{\beta F_{eq} S_1}{1 + F_{eq}} - \frac{\beta S_{eq} F_1}{\left( 1 + F_{eq} \right) ^2} -(\mu + r) S_1 + k_R R_1 \nonumber \\ \frac{\partial I_{L1}}{\partial t}= & {} \sigma \nabla ^2 I_{L1} + \frac{\beta F_{eq} S_1}{1 + F_{eq}} + \frac{\beta S_{eq} F_1}{\left( 1 + F_{eq} \right) ^2} - \left( \theta _L + \gamma \right) I_{L1} \nonumber \\ \frac{\partial I_{H1}}{\partial t}= & {} \sigma \nabla ^2 I_{H1} + \gamma I_{L1} - \theta _{H} I_{H1} \nonumber \\ \frac{\partial R_1}{\partial t}= & {} \sigma \nabla ^2 R_1 + \left( S_1 + I_{L1} + I_{H1} \right) r - \left( \mu + k_R \right) R_1 \nonumber \\ \frac{\partial F_1}{\partial t}= & {} \left( I_{L1} + I_{H1} \right) f - \mu _F F_1 \end{aligned}$$

in which we have made use of the two constants in (11).

Since these Eq. (26) are to be solved in the rectangular domain $-L< x < L$, $-B< y < B$, subject to Neumann boundary conditions of the form in (7), (8), their solutions are sought in the Fourier-series forms

27 $$\begin{aligned} S_1 (x,y,t)= & {} \sum _{m=0}^{\infty } \sum _{n=0}^{\infty } S^L_{m,n} (t) \cos \left( \frac{m\pi (x-L)}{2L} \right) \cos \left( \frac{n\pi (y-B)}{2B} \right) \nonumber \\ I_{L1} (x,y,t)= & {} \sum _{m=0}^{\infty } \sum _{n=0}^{\infty } E^L_{m,n} (t) \cos \left( \frac{m\pi (x-L)}{2L} \right) \cos \left( \frac{n\pi (y-B)}{2B} \right) \nonumber \\ I_{H1} (x,y,t)= & {} \sum _{m=0}^{\infty } \sum _{n=0}^{\infty } H^L_{m,n} (t) \cos \left( \frac{m\pi (x-L)}{2L} \right) \cos \left( \frac{n\pi (y-B)}{2B} \right) \nonumber \\ R_1 (x,y,t)= & {} \sum _{m=0}^{\infty } \sum _{n=0}^{\infty } R^L_{m,n} (t) \cos \left( \frac{m\pi (x-L)}{2L} \right) \cos \left( \frac{n\pi (y-B)}{2B} \right) \nonumber \\ F_1 (x,y,t)= & {} \sum _{m=0}^{\infty } \sum _{n=0}^{\infty } F^L_{m,n} (t) \cos \left( \frac{m\pi (x-L)}{2L} \right) \cos \left( \frac{n\pi (y-B)}{2B} \right) . \end{aligned}$$

The aim is now to calculate the time-dependent Fourier coefficients $S^L_{m,n} (t)$, and so on, for this linearized approximation.

The assumed forms (27) for the solution are substituted into the linearized equations (26). Each equation is then multiplied by a typical basis function

28 $$\begin{aligned} \cos \left( k\pi (x-L) / (2L) \right) \cos \left( \ell \pi (y-B) / (2B) \right) , \end{aligned}$$

for integers $k = 0, 1, 2, \dots$, $\ell = 0, 1, 2, \dots$, and integrated over the rectangular solution domain, making use of the orthogonality of the trigonometric functions. Since the partial differential equations are linear and have constant coefficients, each Fourier mode $(k, \ell )$ de-couples from all the others, and after some algebra, the Fourier-analyzed partial differential equations (26) yield a system of five ordinary differential equations of the form

29 $$\begin{aligned} \frac{\textrm{d} \textbf{V}^L_{k,\ell }}{\textrm{d} t} = \textbf{J}_{k,\ell } \textbf{V}^L_{k,\ell } \end{aligned}$$

independently for each mode $(k,\ell )$. The system involves the $(5 \times 1)$ vector

30 $$\begin{aligned} \textbf{V}^L_{k,\ell } = \bigl [ S^L_{k,\ell } (t); E^L_{k,\ell } (t); H^L_{k,\ell } (t); R^L_{k,\ell } (t); F^L_{k,\ell } (t) \bigr ]^T \end{aligned}$$

which consists of all the time-dependent Fourier coefficients that have been defined in the assumed solution (27). It also involves the $(5 \times 5)$ Jacobian matrix

31 $$\begin{aligned} \textbf{J}_{k,\ell } = \left[ \begin{array}{c c c c c} -A_{k,\ell } &{}\quad \Gamma _{eq} &{}\quad \Gamma _{eq} &{}\quad \Gamma _{eq}+k_R &{}\quad -Q_{eq} \\ P_{eq} &{}\quad -B_{k,\ell } &{}\quad 0 &{}\quad 0 &{}\quad Q_{eq} \\ 0 &{}\quad \gamma &{}\quad -C_{k,\ell } &{}\quad 0 &{}\quad 0 \\ r &{}\quad r &{}\quad r &{}\quad -D_{k,\ell } &{}\quad 0 \\ 0 &{}\quad f &{}\quad f &{}\quad 0 &{}\quad -\mu _F \end{array} \right] \end{aligned}$$

in which, for ease of display, we have introduced seven additional intermediate constants. These are

32 $$\begin{aligned} \Gamma _{eq} = b \left( 1 - 2 N_{eq} \right) ;\quad P_{eq} = \frac{\beta F_{eq}}{1 + F_{eq}};\quad Q_{eq} = \frac{\beta S_{eq}}{\left( 1 + F_{eq} \right) ^2} \end{aligned}$$

and

33 $$\begin{aligned} A_{k,\ell }= & {} \sigma \Delta _{k,\ell }^2 - \Gamma _{eq} + P_{eq} + \mu + r \nonumber \\ B_{k,\ell }= & {} \sigma \Delta _{k,\ell }^2 + \theta _L + \gamma \nonumber \\ C_{k,\ell }= & {} \sigma \Delta _{k,\ell }^2 + \theta _H \nonumber \\ D_{k,\ell }= & {} \sigma \Delta _{k,\ell }^2 + \mu + k_R. \end{aligned}$$

The extra constant

34 $$\begin{aligned} \Delta _{k,\ell }^2 = \left( \frac{k\pi }{2L} \right) ^2 + \left( \frac{\ell \pi }{2B} \right) ^2 \end{aligned}$$

used in (33) is an effective squared total wavenumber for the $(k,\ell )$ Fourier mode.

The solution of the vector differential equation (29) can be written down at once. It is

35 $$\begin{aligned} \textbf{V}_{k,\ell }^L (t) = \sum _{q=1}^5 \mathcal {C}_{k,\ell }^{(q)} \exp \left[ \lambda _{k,\ell }^{(q)} t \right] \textbf{v}_{k,\ell }^{(q)} \end{aligned}$$

in which $\lambda _{k,\ell }^{(q)}$ and $\textbf{v}_{k,\ell }^{(q)}$ are the five eigenvalues and their corresponding eigenvectors, for the Jacobian matrix $\textbf{J}_{k,\ell }$ in (31), at each of the Fourier modes $(k,\ell )$. The five constants $\mathcal {C}_{k,\ell }^{(q)}$, $q = 1, \dots , 5$, are so far arbitrary, but their values are determined from the initial values of the Fourier coefficients $S^L_{k,\ell } (0)$ and so on in the vector $\textbf{V}^L_{k,\ell } (0)$ in (30). If, for each Fourier mode $(k,\ell )$, these initial values are given, then the five constants are found by solving the matrix equation

36 $$\begin{aligned} \textbf{Q}_{k,\ell } \textbf{C}_{k,\ell } = \textbf{V}^L_{k,\ell } (0) \end{aligned}$$

for the vector

$$\begin{aligned} \textbf{C}_{k,\ell } = \left[ \mathcal {C}_{k,\ell }^{(1)}; \dots ; \mathcal {C}_{k,\ell }^{(5)} \right] ^T. \end{aligned}$$

The $$(5 \times 5)$$ matrix

$$\begin{aligned} \textbf{Q}_{k,\ell } = \left[ \textbf{v}_{k,\ell }^{(1)}; \dots ; \textbf{v}_{k,\ell }^{(5)} \right] \end{aligned}$$

in the initial condition (36) is made up of the five eigenvectors of the Jacobian matrix (31).

In principle, this analysis now gives the full linearized solution in some neighbourhood of one of the equilibrium points in Sect. 3. All that is required to evaluate the solution is to specify the initial condition $\textbf{V}^L_{k,\ell } (0)$ in (30), for each Fourier mode. The eigenvalues $\lambda _{k,\ell }^{(q)}$ then determine whether the equilibrium state is stable and small perturbations to equilibrium conditions die away with time, or unstable such that the linearized solution grows exponentially with time.

Calculating these eigenvalues for general parameter values is, however, extremely difficult. Consequently, it is often not possible to give precise conditions under which the three feasible steady states (15), (16), and (24) with the plus sign chosen, are stable or unstable. In those cases, the eigenvalues and eigenvectors must be evaluated numerically, and the solutions (35) then obtained for Fourier modes $k, \ell = 0, 1, 2, \dots$. Finally, the linearized population distributions are evaluated approximately from the Fourier series (27).

In spite of these difficulties, however, it turns out that some important analytical information is nevertheless able to be obtained about the stability of these steady states. Firstly, the $(5 \times 5)$ Jacobian matrix $\textbf{J}_{k,\ell }$ in (31) simplifies very considerably when applied to the total extinction steady state (15), and its eigenvalues can, in fact, be calculated in closed form. After some algebra, they turn out to be

37 $$\begin{aligned}{} & {} \lambda _{k,\ell }^{(1)} = - \mu _F; \quad \lambda _{k,\ell }^{(2)} = - B_{k,\ell }; \quad \lambda _{k,\ell }^{(3)} = - C_{k,\ell }; \nonumber \\{} & {} \lambda _{k,\ell }^{(4)} = - \sigma \Delta _{k,\ell }^2 + \left( b - \mu \right) ; \quad \lambda _{k,\ell }^{(5)} = - \sigma \Delta _{k,\ell }^2 - \left( \mu + k_R + r \right) . \end{aligned}$$

The auxiliary constants in these expressions are as defined in (33) and (34). Now for high modes, in which integers *k* and $\ell$ are arbitrarily large, these eigenvalues (37) are all real and negative, and so the high Fourier modes are all stable. The critical case is the zeroth mode $k = \ell = 0$ corresponding to a spatially uniform disturbance, when the fourth eigenvalue in the system (37) becomes

$$\begin{aligned} \lambda _{0,0}^{(4)} = \left( b - \mu \right) . \end{aligned}$$

This is positive when birth rate *b* exceeds the death rate $\mu$, so this zeroth mode is unstable. Consequently, the total extinction steady state (15) is unstable.

The mite-free steady state (16) is obviously the desired equilibrium configuration in this model, and so it is important to know under what conditions the linearized solution would predict it to be stable. Unfortunately, this information seems too difficult to obtain exactly, for general parameter values. However, some important information about its stability *can* be derived analytically in the special case when the additional death rates for the lightly and heavily infected wombats are equal, $\mu _L = \mu _H$. In this case, it follows from (11) that $\theta _L = \theta _H$. Then the eigenvalues of the Jacobian matrix (31) evaluated for the mite-free case (16) can, in fact, be calculated exactly. A similar situation was encountered by Beeton et al. (2019). After considerable algebra,^1^ the five eigenvalues of (31) for the mite-free state with $\mu _L = \mu _H$ are found to be

38 $$\begin{aligned} \lambda _{k,\ell }^{(1)}= & {} - \sigma \Delta _{k,\ell }^2 - \gamma - \theta _H \nonumber \\ \lambda _{k,\ell }^{(2)}= & {} - \sigma \Delta _{k,\ell }^2 - \left( b - \mu \right) \nonumber \\ \lambda _{k,\ell }^{(3)}= & {} - \sigma \Delta _{k,\ell }^2 - \mu - k_R - r \nonumber \\ \lambda _{k,\ell }^{(4,5)}= & {} \frac{1}{2} \left\{ - \left( C_{k,\ell } + \mu _F \right) \pm \sqrt{ \left( C_{k,\ell } + \mu _F \right) ^2 - 4 \left[ \mu _F C_{k,\ell } - \frac{f \beta (b - \mu )}{b K} \right] } \right\} . \nonumber \\ \end{aligned}$$

For $b > \mu$, the first three eigenvalues in (38) are all negative and the remaining two eigenvalues $\lambda _{k,\ell }^{(4,5)}$ are both real. This mite-free equilibrium, with $\mu _L = \mu _H$ is stable *if and only if* all these eigenvalues are negative, and this is guaranteed if

$$\begin{aligned} \mu _F C_{k,\ell } - \frac{f \beta (b - \mu )}{b K} > 0 \end{aligned}$$

for all spatial Fourier modes $k, \ell = 0, 1, 2, \dots$. This inequality is trivially true for high modes *k* and $\ell$, but again, the zeroth modes $k = \ell = 0$ pose the greatest challenge. Setting both *k* and $\ell$ to zero in this inequality and rearranging then gives the stability result

### Theorem 1

If the death rates are equal across the two infected classes $I_L$ and $I_H$, that is, $\mu _L = \mu _H$, the mite-free steady state (16) is **stable** if and only if

39 $$\begin{aligned} \mu _F > \frac{f \beta \left( b- \mu \right) \left( \mu + k_R \right) }{b \left( \mu + k_R + r \right) \left( \mu + \mu _H + r \right) }. \end{aligned}$$

**Fig. 3:**
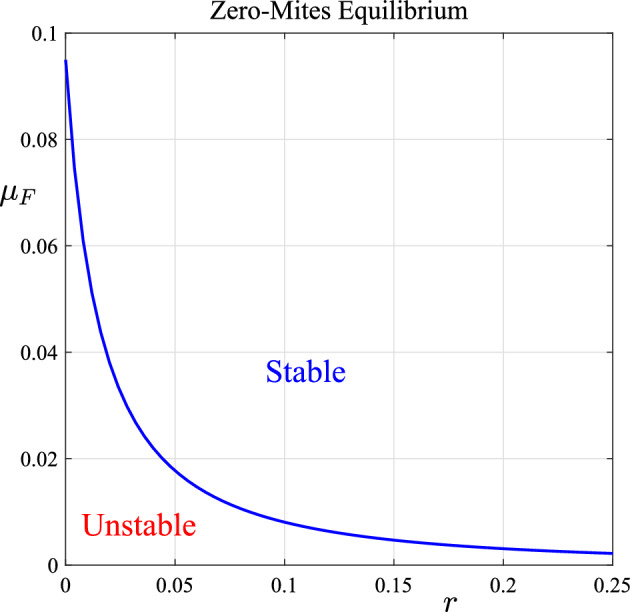
Region of stability for the mite-free equilibrium, as predicted by the linearized solution for $\mu _L = \mu _H$ and parameter values taken from Table 1

This condition (39) was found also by Beeton et al. (2019) for the purely time-dependent model. Although this stability condition (39) is only strictly true for $\mu _L = \mu _H$, we anticipate that it will give at least an approximate guide to the stability of this key mite-free state more generally, and so the region of stability predicted by (39) is shown in Fig. 3 for parameter values taken from Table 1. When stability condition (39) does not hold, then the endemic state (24) will become the stable situation.

To determine stability of all the equilibrium states more generally, including cases when Theorem 1 does not apply, the linearized solution and its eigenvalues must be determined numerically, and this is now briefly outlined. To fix ideas, let us suppose that all the populations are at their steady-state values $\left( S_{eq}, I_{L,eq}, I_{H,eq}, R_{eq}, N_{eq} \right)$. Then, at the initial time $t = 0$, mites are suddenly introduced over some rectangular region within the overall domain; let us suppose that the centre of this mite-infested region is $(x,y) = \left( x_C, y_C \right)$ and the region has size $2 x_R$ by $2 y_R$. Therefore the region newly infested with mites lies over the rectangle $x_C - x_R< x < x_C + x_R$, $y_C - y_R< y < y_C + y_R$ and to ensure this lies wholly within our overall domain, we impose conditions $x_R - L< x_C < L - x_R$ and $y_R - B< y_C < B - y_R$. For simplicity, we will assume that the mite population within the infected rectangular region has the constant value $\varepsilon$. Consequently, the initial condition on the mites is

40 $$\begin{aligned} F(x,y,0) = F_{eq} + {\left\{ \begin{array}{ll} \varepsilon , &{} \quad \text {inside rectangle} \\ 0, &{} \quad \text {outside rectangle}. \end{array}\right. } \end{aligned}$$

(We observe that Eq. (40) now serves to *define* the small parameter $\varepsilon$.) This condition may now be used to determine the initial values $F^L_{m,n} (0)$ of the Fourier series for the mite numbers, in the expression (27). This series representation is subject to Fourier analysis, by multiplying by the basis functions (28) and integrating over the entire domain $-L< x < L$, $-B< y < B$. A somewhat lengthy calculation yields

41 $$\begin{aligned} F^L_{0,0} (0)= & {} \left( \frac{x_R}{L} \right) \left( \frac{y_R}{B} \right) \nonumber \\ F^L_{0,\ell } (0)= & {} \frac{4}{\ell \pi } \left( \frac{x_R}{L} \right) \cos \left( \frac{\ell \pi \left( B - y_C \right) }{2B} \right) \sin \left( \frac{\ell \pi y_R}{2B} \right) , \quad \ell > 0 \end{aligned}$$

for the $m=0$ modes, along with

42 $$\begin{aligned} F^L_{k,0} (0) = \frac{4}{k\pi } \left( \frac{y_R}{B} \right) \cos \left( \frac{k\pi \left( L - x_C \right) }{2L} \right) \sin \left( \frac{k\pi x_R}{2L} \right) \quad k > 0 \end{aligned}$$

for the modes with $n=0$. Finally, the general case gives

43 $$\begin{aligned} F^L_{k,\ell } (0)&{=}&\frac{16}{k\ell \pi ^2} \cos \left( \frac{k\pi \left( L {-} x_C \right) }{2L} \right) \sin \left( \frac{k\pi x_R}{2L} \right) \cos \left( \frac{\ell \pi \left( B {-} y_C \right) }{2B} \right) \sin \left( \frac{\ell \pi y_R}{2B} \right) \nonumber \\{} & {} \quad \text {for } \quad k = 1, 2, 3, \dots , \quad \ell = 1, 2, 3, \dots . \end{aligned}$$

Because the solution is starting from an equilibrium population that has been perturbed by the introduction of mites at $t=0$, the initial vector $\textbf{V}^L_{k,\ell }$ in (30) becomes simply

44 $$\begin{aligned} \textbf{V}^L_{k,\ell } = \bigl [ 0; 0; 0; 0; F^L_{k,\ell } (0) \bigr ]^T. \end{aligned}$$

Furthermore, because the approximate system in this Section is linear (with constant coefficients), then each Fourier mode $\left( k, \ell \right)$ acts completely independently of all the other modes, as discussed above. Consequently, at each separate mode $\left( k, \ell \right)$, the $( 5 \times 1)$ matrix equation (36) can be solved for the five constants in the vector $\textbf{C}_{k,\ell }$ using the initial conditions (44), with values for $F^L_{k,\ell } (0)$ taken from whichever of the expressions (41)–(43) is appropriate for the Fourier mode under consideration. The solution (27) can now be evaluated at any desired time, using the formula (35) for its time-dependent Fourier coefficients.

In practice, we choose to smooth the discontinuous initial condition (40), since it is well known that Fourier series of the type (27) cannot converge near a discontinuity; instead, the reconstructed function contains undesirable oscillations in that vicinity. This is known as the Gibbs phenomenon (Kreyszig 2011, page 515), and can be a particular problem in population models in which a population drops to zero, since spurious small oscillations might then produce negative population values instead of exactly zero, and this of course is unphysical. To avoid this, we use Lanczos smoothing (see Duchon 1979). To illustrate this for a single independent variable *x*, consider a periodic function *f*(*x*) over $-L< x < L$ represented spectrally as

$$\begin{aligned} f(x) = \sum _{m=0}^{\infty } A_m \cos \left( m\pi (x-L) / (2L) \right) . \end{aligned}$$

If *f*(*x*) has a discontinuity at some point $x_D \in (-L, L)$, this series will produce oscillations near $x_D$ according to the Gibbs phenomenon. To avoid this, the Fourier coefficients $A_m$ are replaced with new coefficients

$$\begin{aligned} \overline{A}_m = A_m \frac{\sin \left( \sigma _L m \right) }{\sigma _L m}. \end{aligned}$$

This replaces the original discontinuous function *f*(*x*) with a new function which can be shown to be the average of *f*(*x*) over a moving window $\left( x-\sigma _L, x+\sigma _L \right)$ centred at the point *x*. For appropriate choices of the Lanczos parameter $\sigma _L$, the new function so produced closely approximates the original function except that the discontinuity is replaced by a rapidly-varying smooth change without overshoot. In practice, we choose the Lanczos parameter to be about $\sigma _L \approx 0.05$.

## Numerical solution of nonlinear problem

We now seek a numerical solution to the full system (1)–(5) of nonlinear partial differential equations in Sect. 2, that can describe large-amplitude deviations from the equilibrium populations discussed in Sect. 3. We make use of a spectral method, based on insights gained from the linearized solution (25), (27) in Sect. 4, and now seek a numerical solution to the nonlinear equations in the truncated Fourier-series form

45 $$\begin{aligned} S(x,y,t)= & {} \sum _{m=0}^M \sum _{n=0}^N S_{m,n} (t) \cos \left( \frac{m\pi (x-L)}{2L} \right) \cos \left( \frac{n\pi (y-B)}{2B} \right) \nonumber \\ I_L (x,y,t)= & {} \sum _{m=0}^M \sum _{n=0}^N E_{m,n} (t) \cos \left( \frac{m\pi (x-L)}{2L} \right) \cos \left( \frac{n\pi (y-B)}{2B} \right) \nonumber \\ I_H (x,y,t)= & {} \sum _{m=0}^M \sum _{n=0}^N H_{m,n} (t) \cos \left( \frac{m\pi (x-L)}{2L} \right) \cos \left( \frac{n\pi (y-B)}{2B} \right) \nonumber \\ R(x,y,t)= & {} \sum _{m=0}^M \sum _{n=0}^N R_{m,n} (t) \cos \left( \frac{m\pi (x-L)}{2L} \right) \cos \left( \frac{n\pi (y-B)}{2B} \right) \nonumber \\ F(x,y,t)= & {} \sum _{m=0}^M \sum _{n=0}^N F_{m,n} (t) \cos \left( \frac{m\pi (x-L)}{2L} \right) \cos \left( \frac{n\pi (y-B)}{2B} \right) \end{aligned}$$

In this representation, the integers *M* and *N* should be taken as large as possible. These forms are chosen to satisfy the boundary conditions (7) and (8) identically.

The aim of the numerical solution approach is to find the sets of Fourier coefficients $S_{m,n} (t), \dots , F_{m,n} (t)$ in the representation (45). To do this, these Fourier series are substituted into the full nonlinear set (1)–(5), then multiplied by the same sets of basis functions (28) that were used for the linearized solution in Sect. 4. In this case, however, the integers in these basis functions only take values $k = 0, 1, 2, \dots , M$, $\ell = 0, 1, 2, \dots , N$, since the series in the representations (45) are truncated to total orders *M* and *N*. The resulting equations are now integrated over the solution domain $-L<x < L$, $-B< y < B$ as before, making use of the orthogonality of the cosine functions.

Unlike the linearized solution of Sect. 4, where all the Fourier modes de-coupled and so could be treated independently of each other, the nonlinearity of the full system of equations causes each Fourier coefficient to be affected by all the others in the system. In addition, here we also want to allow the treatment rate *r* to be a function *r*(*x*, *y*, *t*) of both position and time. Consequently, the Fourier decomposition of the system (1)–(5) now gives rise to a large coupled nonlinear system, which we present here for completeness. The susceptible sub-population yields

46 $$\begin{aligned} \frac{\textrm{d} S_{k,\ell } (t)}{\textrm{d} t}= & {} \frac{1}{\delta _{k,\ell } LB} \int _{-B}^{B} \int _{-L}^{L} \biggl [ bN(1 - N) - \frac{\beta SF}{1+F} - rS \biggr ] \nonumber \\{} & {} \quad \times \cos \left( \frac{k\pi (x-L)}{2L} \right) \cos \left( \frac{\ell \pi (y-B)}{2B} \right) \, \textrm{d}x \, \textrm{d}y \nonumber \\{} & {} \quad - \sigma \Delta _{k,\ell }^2 S_{k,\ell } (t) - \mu S_{k,\ell } (t) + k_R R_{k,\ell } (t) \end{aligned}$$

and the lightly infected (“exposed”) group results in the system of ordinary differential equations

47 $$\begin{aligned} \frac{\textrm{d} E_{k,\ell } (t)}{\textrm{d} t}= & {} \frac{1}{\delta _{k,\ell } LB} \int _{-B}^{B} \int _{-L}^{L} \biggl [ \frac{\beta SF}{1+F} - r I_L \biggr ] \nonumber \\{} & {} \quad \times \cos \left( \frac{k\pi (x-L)}{2L} \right) \cos \left( \frac{\ell \pi (y-B)}{2B} \right) \, \textrm{d}x \, \textrm{d}y \nonumber \\{} & {} \quad - \sigma \Delta _{k,\ell }^2 E_{k,\ell } (t) - \left( \mu + \gamma + \mu _L \right) E_{k,\ell } (t). \end{aligned}$$

The heavily infected sub-population of wombats is modelled by (3), which after Fourier analysis gives rise to

$$\begin{aligned} \frac{\textrm{d} H_{k,\ell } (t)}{\textrm{d} t}= & {} - \frac{1}{\delta _{k,\ell } LB} \int _{-B}^{B} \int _{-L}^{L} r I_H \cos \left( \frac{k\pi (x-L)}{2L} \right) \cos \left( \frac{\ell \pi (y-B)}{2B} \right) \, \textrm{d}x \, \textrm{d}y \\{} & {} \quad - \sigma \Delta _{k,\ell }^2 H_{k,\ell } (t) + \gamma E_{k,\ell } (t) - \left( \mu + \mu _H \right) H_{k,\ell } (t) \end{aligned}$$

and the equation for the recovered wombats yields a further system of differential equations

48 $$\begin{aligned} \frac{\textrm{d} R_{k,\ell } (t)}{\textrm{d} t}= & {} \frac{1}{\delta _{k,\ell } LB} \int _{-B}^{B} \int _{-L}^{L} r \left[ S + I_L + I_H \right] \nonumber \\{} & {} \quad \times \cos \left( \frac{k\pi (x-L)}{2L} \right) \cos \left( \frac{\ell \pi (y-B)}{2B} \right) \, \textrm{d}x \, \textrm{d}y \nonumber \\{} & {} \quad - \sigma \Delta _{k,\ell }^2 R_{k,\ell } (t) - \left( \mu + k_R \right) R_{k,\ell } (t). \end{aligned}$$

The final partial differential equation (5) for the mite population results in the system

49 $$\begin{aligned} \frac{\textrm{d} F_{k,\ell } (t)}{\textrm{d} t} = f \left[ E_{k,\ell } (t) + H_{k,\ell } (t) \right] - \mu _F F_{k,\ell } (t). \end{aligned}$$

These equations apply to all the Fourier modes $k = 0, 1, 2, \dots , M$, $\ell = 0, 1, 2, \dots , N$ in the truncated series representation (45).

The constants $\Delta _{k,\ell }^2$ appearing in the system (46)–(49) are the same as those in (34) used in the linearized solution of Sect. 4. For convenience of notation, we have also defined constants

50 $$\begin{aligned} \delta _{k,\ell } = \left\{ \begin{array}{ll} 4, &{}\quad \text {if } \quad k=0 \text { and } \ell = 0 \\ 2, &{}\quad \text {if either } \quad k=0 \text { or } \ell = 0 \\ 1, &{}\quad \text {if }\quad k \ne 0 \text { and } \ell \ne 0 \end{array} \right. \end{aligned}$$

in this governing system of equations for the Fourier coefficients.

These relations (46)–(49) constitute a system of $5(M+1)(N+1)$ coupled nonlinear ordinary differential equations for the sets of coefficients $S_{k,\ell }$, $E_{k,\ell }$, $H_{k,\ell }$, $R_{k,\ell }$ and $F_{k,\ell }$ for $k = 0, 1, 2, \dots , M$ and $\ell = 0, 1, 2, \dots , N$. We use the numerical integration routine ode45 provided in the programming language matlab to integrate these equations forward in time, starting from appropriate initial conditions, which are chosen to mimic those used for the linearized solution in Sect. 4. That is, all the coefficients $S_{k,\ell } (0)$ and so on are set to zero at time $t = 0$, so that the solution starts from a chosen equilibrium state, except for the coefficients $F_{k,\ell } (0)$ for the initial number density of mites. These are chosen to be the same as for the linearized solution, and are given by (41)–(43), so as to generate the initial rectangular deposition of mites described by (40), although in practice we again smooth this discontinuous initial mite distribution using the Lanczos filter, as discussed at the end of Sect. 4. Once these coefficients $S_{k,\ell } (t)$ and so on have been determined, by integration of the system (46)–(49), the populations of wombats and mites are then reconstructed from their Fourier-series representations (45).

## Presentation of results

### Comparison of linearized and nonlinear solutions

**Fig. 4:**
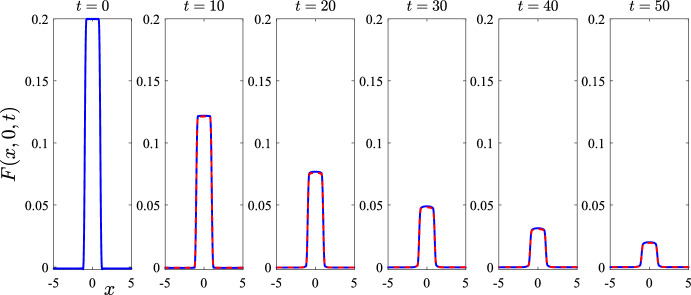
A comparison of results obtained from the linearized solution of Sect. 4 with those obtained using the algorithm in Sect. 5 for the nonlinear problem. The parameters are all as given in Table 1. This figure illustrates the mite numbers *F* at the initial time $t=0$ and five subsequent times $t = 10$, 20, 30, 40 and 50, on the centre-plane $y=0$. Linearized results are shown with solid (blue online) curves and numerical nonlinear results are drawn with dashed (red) lines (color figure online)

We begin this presentation of results by checking the agreement between the numerical algorithm of Sect. 5 with the exact solution for the linearized problem in Sect. 4. To do this, we take the parameters in the model to be those given in Table 1; the solution is started at the mite-free equilibrium (16), except that mites are added according to the initial condition (40) with maximum mite numbers $\varepsilon = 0.2$. For simplicity, the rectangle over which the mites are initially introduced into the system is centred at the origin, $\left( x_C, y_C \right) = (0,0)$, with half-lengths $x_R = 1$ and $y_R = 1$ kilometres. (Lanczos smoothing is then applied to the initial mites distribution, as described at the end of Sect. 4). This is a case in which good agreement between the linearized solution (Sect. 4) and the full nonlinear results (Sect. 5) is to be expected, as the mite population diffuses and dies, and the system returns to its mite-free state (16).

Figure 4 shows a centre-line profile of the mite population as a function of distance *x*, for the initial time $t = 0$ and a further five times $t = 10, 20, \dots , 50$ days. These profiles were taken from the linearized solution (27) and the numerical nonlinear solution (45) by evaluating those expressions on the plane $y = 0$. At initial time $t = 0$, the centre-line profile for the mites consists simply of $\varepsilon = 0.2$ mites over the interval $-1< x < 1$, but no mites otherwise, according to the initial condition (40), and this appears in the first diagram at the left of the figure. The nonlinear results presented in this diagram have all been obtained using $(M,N) = (101,101)$ Fourier coefficients in the truncated series (45), and five times that number of numerical mesh points were used in the physical (*x*, *y*) space. This is done so as to ensure that the numerical evaluations of the integrals in the formulae (46)–(48) are all performed to sufficient accuracy. In Fig. 4, the linearized solution is shown with solid lines (blue online) and the nonlinear results are drawn using (red) dashed lines.

The agreement between the linearized and nonlinear solutions for this case is excellent. For the five sample times $t = 10, \dots , 50$ illustrated in Fig. 4, there is no discernible difference between the two sets of results visible in the figure. This agreement continues to hold at later times than shown here, and has been checked carefully. This is an important step in the verification of the nonlinear solution algorithm in Sect. 5, and it gives confidence in our spectral method for solving the full nonlinear equations (1)–(5). For this example, the total wombat number started at the equilibrium value $N_{eq} = 0.8$ in (16) and stayed very close to that value throughout, and so has not been presented here.

**Fig. 5:**
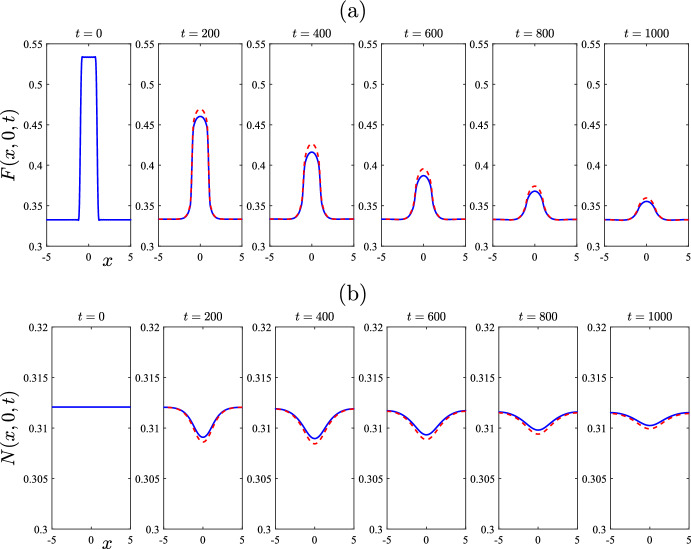
A comparison of results obtained from the linearized solution of Sect. 4 with those obtained using the algorithm in Sect. 5 for the nonlinear problem. The parameters are as given in Table 1, except that $\mu _F = 0.005$ and $r = 0.05$. This figure illustrates: **a** the mite numbers *F*, and **b** the total wombat numbers *N* at the initial time $t=0$ and five subsequent times $t = 200$, 400, 600, 800 and 1000, on the centre-plane $y=0$. The linearized solution is drawn with solid (blue) lines and the nonlinear results with dashed (red) lines (color figure online)

In Fig. 5, we continue this comparison of the predictions of the approximate linearized theory (from Sect. 4) with the numerical solution of the fully nonlinear equations (using the scheme in Sect. 5). In this example, however, we now choose parameter values for which the linearized solution indicates that the “plus” steady state should be the stable one, and accordingly we choose this “plus” state (24) as the initial condition, except that the system is again also perturbed by adding a patch of mites over the same rectangular region $-1< x < 1$, $-1< y < 1$ centred at the origin, as for Fig. 4. Again we assume initial disturbance magnitude $\varepsilon = 0.2$ for the mite numbers in the rectangle.

For the example presented in Fig. 5, the mite death rate has been set at the (admittedly unrealistic) smaller value $\mu _F = 0.005$, so as to explore the dynamical behaviour possible in this system. The treatment rate is also reduced significantly to $r = 0.05$. It can be seen from the diagrams on the right-hand side of Fig. 2 that, at this treatment rate $r = 0.05$, it is to be expected that the endemic equilibrium (24)—with the “plus” sign chosen for the square-root term—will now become the stable steady state, rather than the mite-free case (16). This is indeed confirmed by the linearized solution of Sect. 4 and the stability diagram Fig. 3 . This would be a less desirable situation from the biological point of view, even if it were achievable, since now both the wombats and the mites would survive in the environment, under these changed conditions.

The mite population *F* on the centreline $y = 0$ is shown in Fig. 5a and the total wombat numbers *N* are displayed in part (b). In each figure, the first diagram on the left gives the situation at the initial time $t=0$. Thus the total wombat numbers in part (b) are calculated from the endemic equilibrium (24) for the “plus” branch, giving $N_{eq} = 0.3121$. The steady-state mite numbers with treatment rate $r = 0.05$ are calculated to be $F_{eq} = 0.3337$ and this likewise serves as the initial mite number density, except over the interval $-1< x < 1$ where additional mite numbers $\varepsilon = 0.2$ have been added, to give $F = 0.5337$ in that interval. This perturbed initial situation is shown in Fig. 5a. As expected, the perturbation to the mite numbers decays away as time proceeds, although the decay rate is clearly much slower than that shown in Fig. 4, and a much longer time interval is required, in order to see this effect. Thus, Fig. 5 gives mite and wombat numbers at the five later times $t = 200, 400, \dots , 1000$. Eventually, the mite numbers return to their steady-state value $F_{eq} = 0.3337$ across the entire spatial domain and the wombat sub-populations also return slowly to their equilibrium values. It will be seen from Fig. 5 that, while there is generally good agreement between the linearized solution (sketched with solid lines) and the nonlinear solution (drawn with dashed lines), the agreement between them is not as precise as was the case in Fig. 4. This is not a numerical error on the part of the nonlinear solution, since convergence of the Fourier series in the representation (45) has been monitored very carefully; the results in Fig. 5 have been generated with $(M,N) = (61,61), (81,81)$ and (101, 101) modes, and are indistinguishable from each other. Rather, the slight discrepancies between the linearized approximation and the nonlinear results, near the maxima or minima of the curves shown in Fig. 5, are an indication of the effects of nonlinearity on the spatial profiles, near these crests or troughs.

**Fig. 6:**
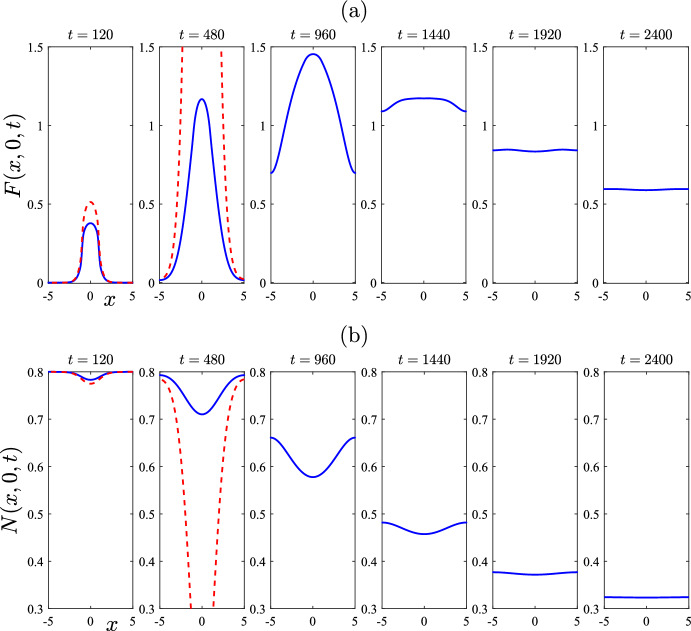
A comparison of results obtained from the linearized solution of Sect. 4 with those obtained using the algorithm in Sect. 5 for the nonlinear problem. The parameters are as given in Table 1, except that $\mu _F = 0.005$ and $r = 0.05$. This figure illustrates: **a** the mite numbers *F*, and **b** the total wombat numbers *N* at times $t=120$ and $t = 480$, 960, 1440, 1920 and 2400 days, on the centre-plane $y=0$. The initial condition consisted of a perturbation $\varepsilon =0.2$ to the mite-free state (16). The linearized solution (dashed, red lines) soon fails, but the nonlinear solution (solid, blue lines) evidently makes a transition to the endemic “plus” steady state (color figure online)

So far, the linearized solution from Sect. 4 has given a reasonably good indication of the behaviour of the fully nonlinear wombat–mite system. This is because we have only used initial conditions that consist of a moderate perturbation to a stable equilibrium state; as time evolves, the perturbations die away and the system returns to equilibrium. This has given us an important check on the reliability of the nonlinear solution algorithm in Sect. 5.

In Fig. 6, however, this situation now changes radically. The parameter values used in this case are exactly the same as for Fig. 5; but the initial condition has been changed to become a perturbation of the mite-free equilibrium (16), rather than of the (stable) equilibrium (24), for the “plus” case. Consequently, the linearized solution now fails after a relatively short time. This is because linearization is only valid when all the populations remain close to an equilibrium state, as indicated in the expression (25). Linearized solutions are very limited in the behaviours they can exhibit, since, as (35) indicates, they can only either decay exponentially in time (to a stable equilibrium) or else grow exponentially (if the nearby equilibrium is unstable). In Fig. 6, the initial condition consisted of a perturbation to the mite-free equilibrium, which is *unstable* for these parameter values; as a result, the only behaviour the linearized solution can develop is for it to increase without bound as time evolves. This is evident in Fig. 6, where there is moderate agreement with the nonlinear solution at the earliest time $t = 120$ shown, but by time $t = 480$ the linearized solution is predicting an unrealistic explosion in mite numbers. We do not show the linearized solution for later times, since it ceases to be of any further value.

By contrast, the nonlinear solution in Fig. 6 reveals some interesting behaviour, of a fundamentally nonlinear nature. At the initial time $t=0$, the mite population is zero throughout the entire region, with the exception of the rectangle $-1<x < 1$, $-1< y < 1$, where it has the perturbed value $\varepsilon = 0.2$. By the earliest time $t = 120$ shown in Fig. 6, the number of mites in this inner rectangle has grown slightly, as is evident in Fig. 6a. The (nonlinear) solution then grows much larger, and by day number $t = 960$ in part (a), it has reached an approximate maximum of about $F = 1.5$ at the centre of the region. After that time, the peak mite numbers begin to decrease; furthermore, the spatial pattern begins to diminish as the mite population distributes itself more and more uniformly across the environment, here represented by the rectangle $-L< x < L$, $-B< y < B$, with $L = B = 5$ kilometres. The mite population in Fig. 6 is evidently making a transition from the initial perturbed mite-free state (16) to the stable endemic “plus” equilibrium state (24), although it makes a very large amplitude excursion as it does so. The effect on the total wombat numbers in Fig. 6b is consistent with that interpretation of these results, since initially those numbers started at the mite-free value $N_{eq} = 0.8$ given in (16), but by the last time $t = 2400$ shown, they were evidently approaching the “plus” equilibrium value $N_{eq} = 0.3121$ calculated from (24).

**Fig. 7:**
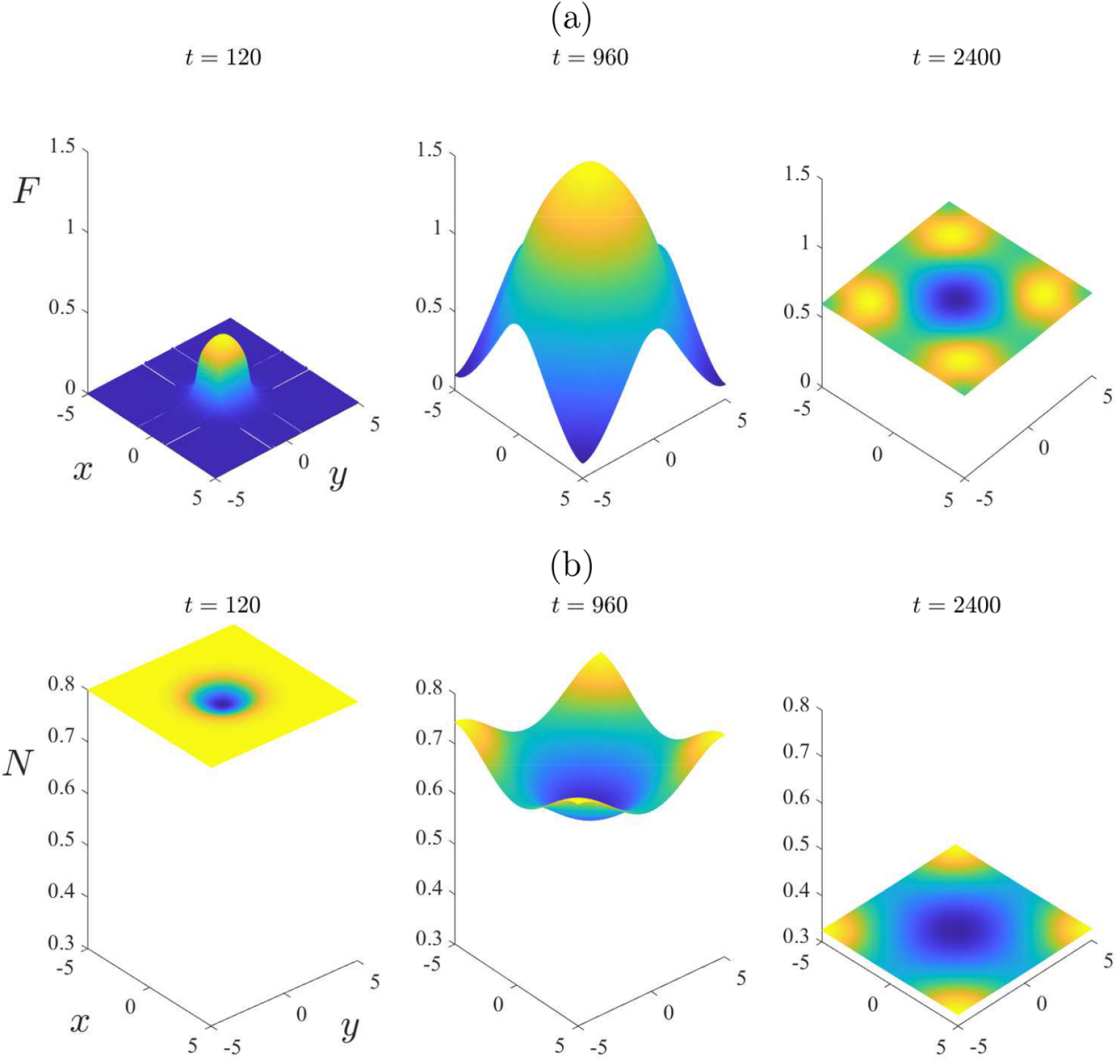
Some spatial patterns for selected times $t=120$, 960 and 2400, for the same case illustrated in Fig. 6. This figure illustrates: **a** the mite numbers *F*, and **b** the total wombat numbers *N*

To illustrate further the role that spatial variability can have in the distribution of mites and wombats, we present in Fig. 7 some spatial patterns at the three selected times $t = 120$, 960 and 2400, for the same case as considered in Fig. 6. At the earliest time $t = 120$, the situation is still moderately close to the initial condition obtained from the mite-free equilibrium (16); thus the initial mite numbers are effectively still zero over the environment, except roughly within the rectangle into which they were introduced with mite density $F = \varepsilon = 0.2$. The total wombat population *N* in part (b) is still close to the initial mite-free value $N_{eq} = 0.8$, although there is a small decrease in wombats within the rectangular region into which the mites were introduced.

The second time $t = 960$ in Fig. 7 corresponds roughly to the moment at which mite numbers reach their maximum at the centre of the region, as shown in Fig. 6. Nevertheless, although mite numbers have risen to about $F = 1.5$ at the centre of the region, at time $t = 960$, they are nevertheless still almost zero at the corners $(x,y) = ( \pm 5, \pm 5)$, as is evident at that time in Fig. 7a. The total wombat numbers in the centre of the region, shown in part (b), have decreased from $N = 0.8$ to about 0.6. After this time the mite numbers decrease slowly, but the wombat numbers become more uniform in space, and decrease very significantly to the point that, by $t = 2400$ days, they are approaching the endemic steady-state value $N_{eq} = 0.3121$.

### Variable treatment rate—nonlinear results

For the example run shown in Figs. 6 and 7, the choice of parameters in the model ensured that the endemic “plus” equilibrium state (24) was the stable one. This means that, as time progresses, the populations within the environment eventually converge to that state. The nonlinear numerical method of Sect. 5 is capable of tracking those changes through time and space, even when the populations make a transition from one equilibrium state to another, as in the example shown. However, as with many dynamical systems of this type, the populations here may experience transitory large-amplitude excursions in which the populations are very far from any of the equilibrium states in Sect. 3, as they make the transition from their initial configuration to their eventual state. This was seen at about time $t = 960$ in Figs. 6 and 7, for example. It is easy to imagine that, in the field, a wildlife manager may notice the development of spatial regions in which mite concentration becomes large, as in Fig. 6a, and may decide to intervene. For wombats in Narawntapu National Park (NNP) in Tasmania, this intervention is accomplished using an insecticide that is introduced onto the wombat skin by means of flaps at the entrances of their underground burrows, as documented by Martin et al. (2019). This treatment runs for about 12 weeks. It is probably not possible, in most cases, to treat the entire environment in this manner, and so the wildlife manager would quite reasonably focus resources on that portion where mite numbers are at their greatest. In this Section, we therefore seek to model how such a strategy alters mite and wombat numbers.

To implement an intervention strategy of this type within our mathematical model, it is now necessary to allow the treatment rate *r* to become a (known) function of both space and time. We do this here by postulating a spatio-temporal function of the type

51 $$\begin{aligned} r(x,y,t) = r_A + \left( r_T - r_A \right) f_T (t) g_T (x,y). \end{aligned}$$

Here, the constant $r_A$ represents a constant “ambient” treatment rate that is applied to the whole environment, perhaps by spraying with a mite growth-retarding hormone, for example. The additional function $f_T (t) g_T (x,y)$ is the extra treatment applied at specific times to targeted sub-regions in space. Since this is only applied over a sub-region within the overall domain, we model the spatial component of the treatment function as

52 $$\begin{aligned} g_T (x,y) = {\left\{ \begin{array}{ll} 1, &{} \quad \text {inside rectangle } \mathcal {R}_T \\ 0, &{} \quad \text {outside rectangle } \mathcal {R}_T \end{array}\right. } \end{aligned}$$

similarly to the form (40) used for the initial condition. Here, the symbol $$\mathcal {R}_T$$ denotes some treatment region, and for simplicity we choose it to be the rectangular region $$| x - x_0 | < x_T$$; $$| y - y_0 | < y_T$$ with centre $$\left( x_0, y_0 \right) $$ and area $$4 x_T y_T $$.

The choice $\mu _F = 0.005$ for the mite death rate used in Figs. 6 and 7 is not biologically reasonable, as observed above, since it would imply that free mites could survive for 200 days, and that is far longer than actually observed. This choice was made only to illustrate the dynamics that can be achieved by a model of this type, since it is possible that related models in slightly altered circumstances might also encounter such behaviour, but in biologically achievable situations [for example, the fungal pathogen causing Bat White Nose syndrome is long lived in the environment (Hoyt et al. 2021)]. For the remainder of this paper, we now focus on parameter values specifically relevant to wombat–mite interactions. These are summarized in Table 1.

**Fig. 8:**
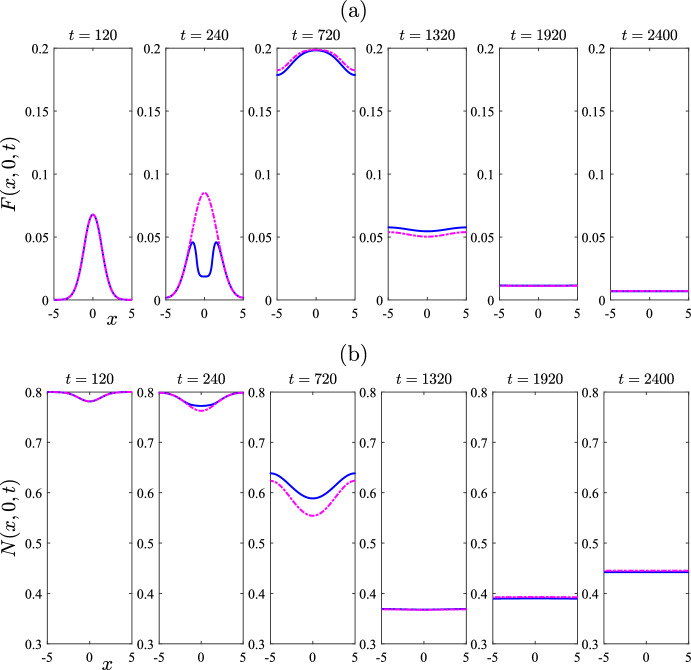
Comparison of treated case (solid line—blue online) with untreated case (chain-dot line—purple online). Results obtained from the algorithm in Sect. 5 for the nonlinear problem. The parameters are as given in Table 1, except that there is assumed to be no ambient treatment, so that $r_A = 0$. There is then a single treatment (at the rate $r = 0.9 / 7$ given in Table 1), over the time interval $200< t < 250$, in the square $-1< x,y < 1$ centred at the origin. This figure illustrates: **a** the mite numbers *F*, and **b** the total wombat numbers *N*, at times $t=120$ and $t = 240$, 720, 1320, 1920 and 2400 days, on the centre-plane $y=0$. The initial condition consisted of a perturbation $\varepsilon =0.2$ to the mite-free state (16) (color figure online)

**Fig. 9:**
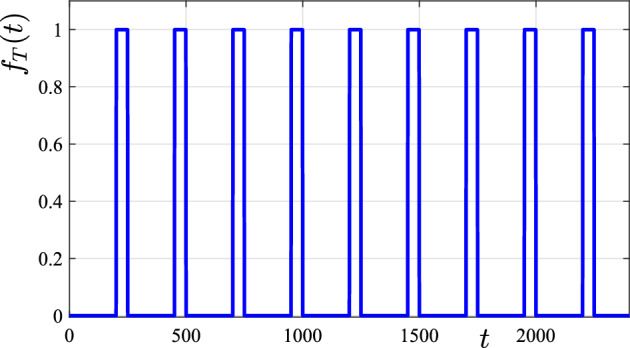
A graph of the switching function $f_T (t)$ described in the text, that allows the treatment to be applied occasionally. Here, treatment is switched on at time $t=200$ days and off again at $t=250$ days. This treatment regime is repeated every 250 days

Figure 8 shows a case in which the system is started from the mite-free equilibrium (16), but with an initial injection of mites over the unit square at the centre of the region, of perturbation magnitude $\varepsilon =0.2$. To ensure the accuracy of the results, the numbers of Fourier modes used were $(M,N) = (121,121)$, with five times those numbers of mesh points. Here, however, a *single* treatment has also been applied at the centre of the physical region where the mite concentration is highest, perhaps mimicking the behaviour of a wildlife manager in the region. The treatment here has been applied over the 50-day interval $200< t < 250$, and over the square region $-1< x < 1$, $-1< y < 1$ in the centre of the wombat environment. Thus the treatment zone $\mathcal {R}_T$ in (52) has parameters $\left( x_0, y_0 \right) = (0,0)$ with $x_T = y_T = 1$. The model parameters are as given in Table 1. In addition, it is assumed in Fig. 8 that there is no ambient treatment over the general environment, so that $r_A = 0$, and the rate within the treatment region has been chosen to be $r_T = 0.9/7 = 0.1286$, as in Table 1. The solid (blue) lines in Fig. 8a, b show, respectively, the number *F* of mites and the total wombat population *N* on the centre-plane $y = 0$, in the case in which this single dose of treatment has been applied, at the six different times indicated on the Figure and in its caption. Also shown in these two diagrams, for comparison, is the case when no treatment at all has been applied, so that $r = 0$ throughout the entire environment. These untreated results are sketched with (purple) chain-dot lines.

Without any treatment, $r = 0$, Fig. 2 shows that the “plus” endemic state, with $F_{eq} = 0.0324$ and $N_{eq} = 0.4663$, is the stable steady-state situation for $\mu _F = 1/19$ (the prevalence of observable mange in the wombat population for this case is $I_{H,eq} / N_{eq} = 1.2$ percent). Thus, although the results were started from a perturbation to the mite-free equilibrium (16), the solution eventually makes a transition to the “plus” endemic state (24). The linearized solution of Sect. 4 is therefore not capable of following this transition between steady states (similar to the situation in Fig. 6), but the nonlinear method of Sect. 5 is able to cope with this strongly nonlinear effect, and the results are shown in Fig. 8 for both the completely untreated case $r=0$ as well as the situation involving the single treatment over the time interval $200< t < 250$.

The use of a single treatment at the centre of the region evidently does not have a great effect on either mite numbers or overall wombat survivability. This is clear from Fig. 8, since after sufficient time has passed, there is almost no difference between the populations with treatment (blue, solid lines) and those without (purple, chain-dot lines). Nevertheless, at the second time $t = 240$ shown in part (a), the treatment has clearly had a strong, short-term effect, since the peak in the mites for the untreated case is replaced by an inverted trough in the case with treatment. It is interesting to observe that, since the solution has been started from an initial condition near the unstable zero-mites equilibrium (16), mite numbers begin to grow exponentially at early times, just as predicted by the linearized solution in Sect. 4, before eventually undergoing nonlinear saturation at about $F = 0.2$ at the time $t = 720$, and then making the transition to the “plus” endemic state for which $F = 0.0324$.

In an attempt to bring about a significant reduction in mite numbers *F*, repeated treatments over the same treatment region $\mathcal {R}_T$ have been investigated, as detailed in (51). This has been achieved in this study by choosing the time-dependent component $f_T (t)$ in (51) to be a switching function that activates at time $t=200$ days and switches off after 50 days at time $t=250$, as in Fig. 8; however, it now reactivates every 250 days after this time. This creates the time-dependent switching function $f_T (t)$ illustrated in Fig. 9 over the total time period $0< t < 2400$ considered here.

**Fig. 10:**
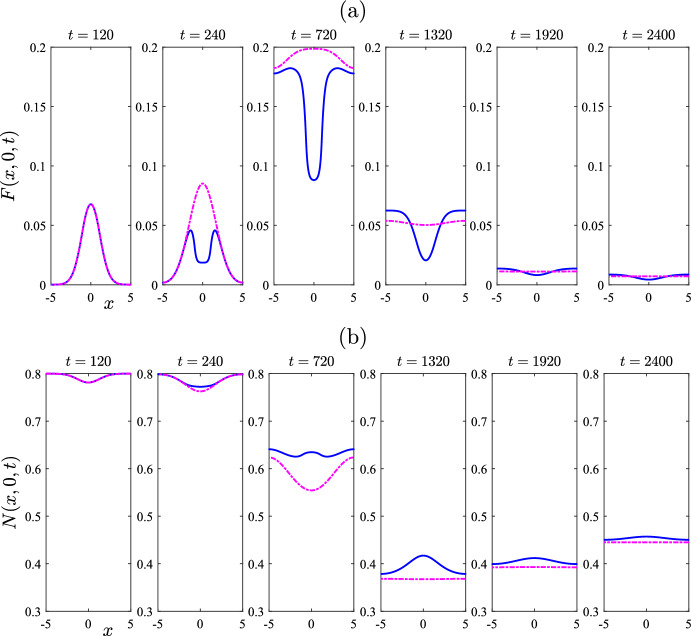
Comparison of treated case (solid line—blue online) with untreated case (chain-dot line—purple online). The parameters are the same as for Fig. 8. This figure illustrates: **a** the mite numbers *F*, and **b** the total wombat numbers *N* at times $t=120$ and $t = 240$, 720, 1320, 1920 and 2400 days, on the centre-plane $y=0$. The initial condition consisted of a perturbation to the mite-free state (16). Multiple treatment occurred over a square centred at the origin $(x,y) = (0,0)$ of the environment, lasting 50 days and repeating every 250 days. There was no ambient treatment, so that $r_A = 0$ with special rate $r_T = 0.9/7$ over the target region (color figure online)

The results of applying this repeated treatment are illustrated in Fig. 10. Here, the parameters are all the same as in Fig. 8, and the treatment region is chosen to be the same square patch $-1< x, y < 1$ located around the centre of the wombat environment. The ambient treatment rate is again $r_A = 0$ and the targeted treatment rate is $r_T = 0.9/7$ over the treatment patch. Now, however, that treatment is repeated periodically in time, as outlined in the schedule shown in Fig. 9. Accuracy was ensured by choosing $M,N = 121,121$ Fourier coefficients in the numerical solution.

It is evident from Fig. 10 that the repeated treatment does indeed reduce the peak mite numbers over the approximate time interval $240< t < 1320$ during which the solution is making its nonlinear transition from the mite-free state (16) to the “plus” endemic state (24). The repeated treatments have resulted in the formation of a “dimple” in the centre-line mite profile *F*(*x*, 0, *t*) in part (a), focussed at the origin. In addition, the overall wombat numbers in part (b) remain slightly above those for the untreated case. However, after sufficient time has passed, only slight advantages remain from the repeated treatment over the centre of the environment.

**Fig. 11:**
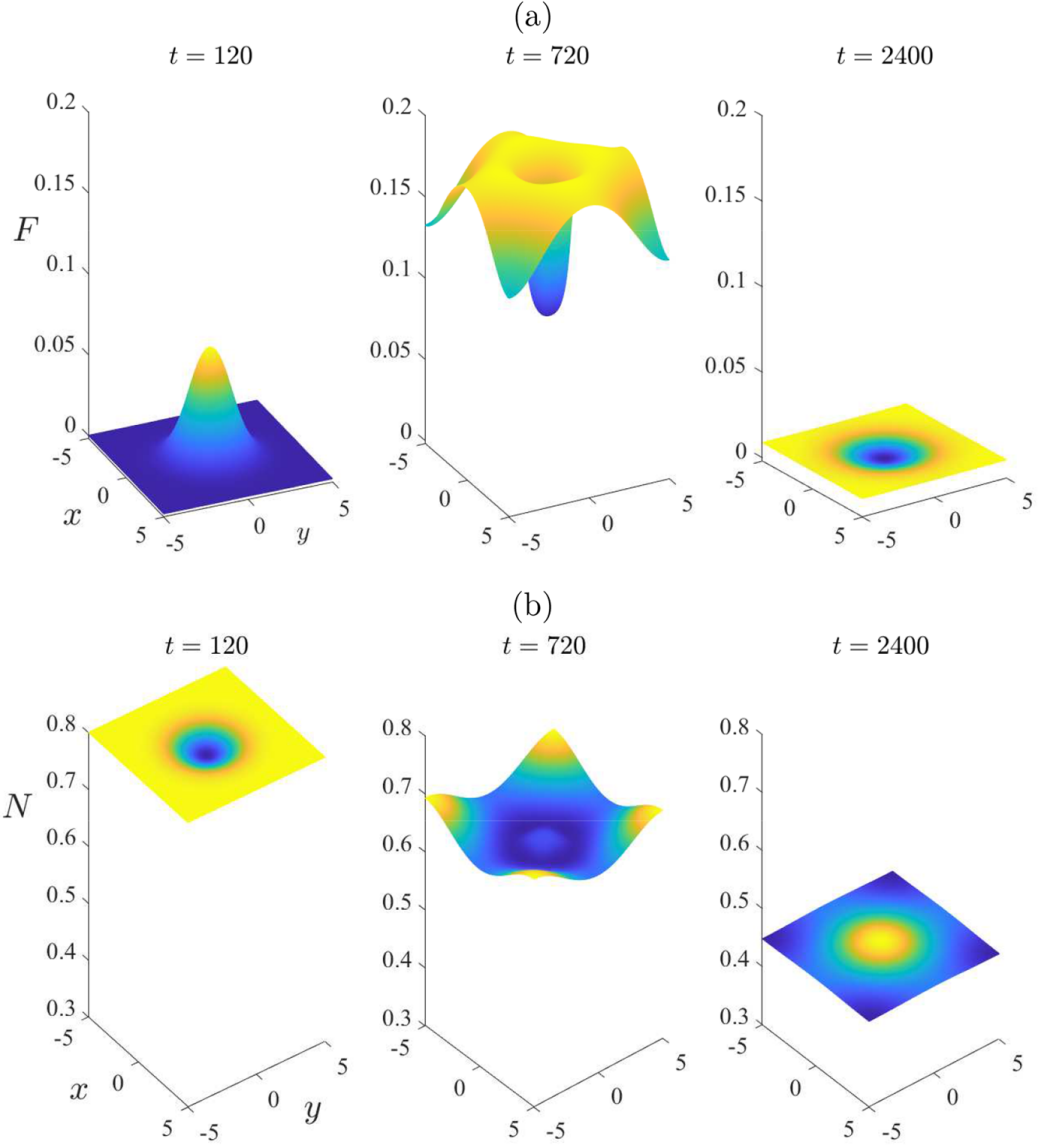
Some spatial patterns for selected times $t=120$, 720 and 2400, for the same case illustrated in Fig. 10 with multiple treatment periods. This figure illustrates: **a** the mite numbers *F*, and **b** The total wombat numbers *N* for the treated case. There was no ambient treatment ($r_A = 0$) and the treatment rate was $r_T = 0.9/7$ over the central target region

Figure 11 illustrates further the way in which the repeated treatment over the rectangular target region $-1< x, y < 1$ has resulted in a dent near the centre of the mite profile. Results are shown at the three times $t = 120$, $t=720$ and $t=2400$ days, and the indented mites profile is clearly evident at the second time $t=720$ in Fig. 11a. Evidently, the repeated application of treatment with the rate $r_T = 0.9/7 = 0.1286$ (taken from Table 1) over the centrally-located square region $-1< x, y < 1$ has resulted in a substantial reduction in mite numbers over this special targeted portion of the environment for times about 720 days. Nevertheless, the long-term benefit of this treatment is fairly minor. This is a disappointing outcome, and suggests that an alternative treatment strategy should be investigated instead.

### Variable treatment rate at a corner

**Fig. 12:**
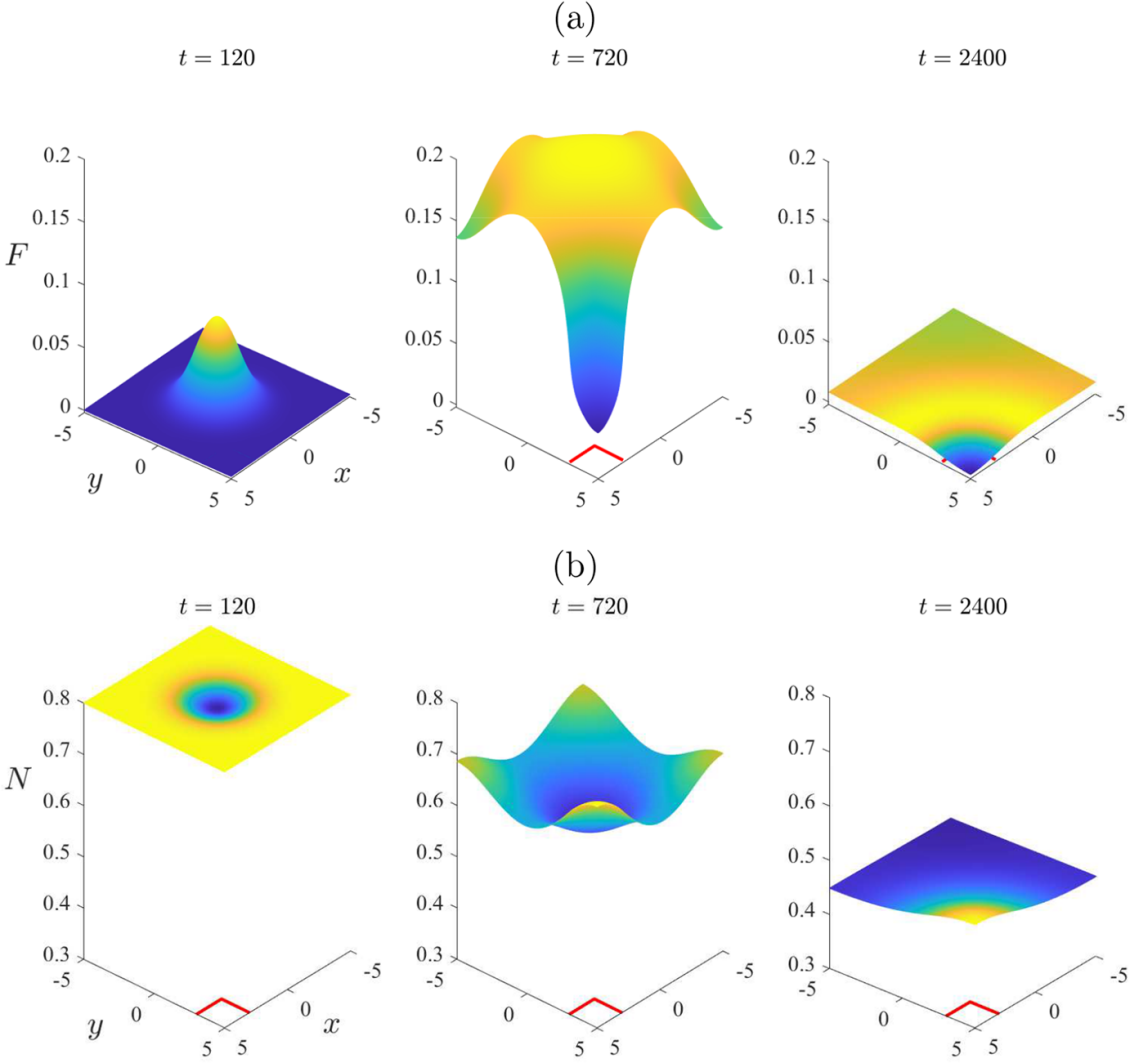
Some spatial patterns for selected times $t=120$, 720 and 2400, for the same (treated) case illustrated in Fig. 10 with multiple treatment periods. There was no ambient treatment ($r_A = 0$) and the treatment rate $r_T = 0.9/7$ was applied over the square corner region indicated with (red) solid lines. This figure illustrates: **a** the mite numbers *F*, and **b** the total wombat numbers *N* (color figure online)

In this model, we have assumed that there is no movement across the boundaries of the spatial environment. For simplicity, we assumed a rectangular environment $-L< x < L$, $-B< y < B$, and the impermeability of these borders was expressed by the boundary conditions (7), (8). These boundaries might physically correspond to rivers, for example, that the wombats are not free to cross. This suggests that it might be possible to protect a group of wombats by moving the targeted treatment zone away from the centre, where the original group of mites was dropped at the initial time $t = 0$ and where mite numbers are consequently greatest, and re-locating it to a corner of the environment.

Accordingly, we consider in Fig. 12 a situation in which the target region has been moved into one corner of the environment. In the notation of condition (52), we have chosen a target region $\mathcal {R}_T$ with centre $\left( x_0, y_0 \right) = (4,4)$ and $x_T = y_T = 1$; this is a square $3< x < 5$, $3< y < 5$ nestled into a corner of the environment, with two sides protected from wombat movement by the conditions (7), (8). To aid understanding, this region is sketched at the bottom of each diagram using a heavy solid line (red online). Figure 12a shows the mite numbers *F* over the entire environment at the three times $t = 120$, 720 and 2400 days, and the total wombat numbers *N* are illustrated in part (b) at those same times. These results were generated using $(M,N) = (121,121)$ Fourier coefficients.

It can be seen, particularly from the last diagram at time $t=2400$ in part (a), that mite numbers have been very substantially reduced over the target region, even although there is not much change over the rest of the environment. The total wombat numbers shown in part (b) also rise slightly in that region.

**Fig. 13:**
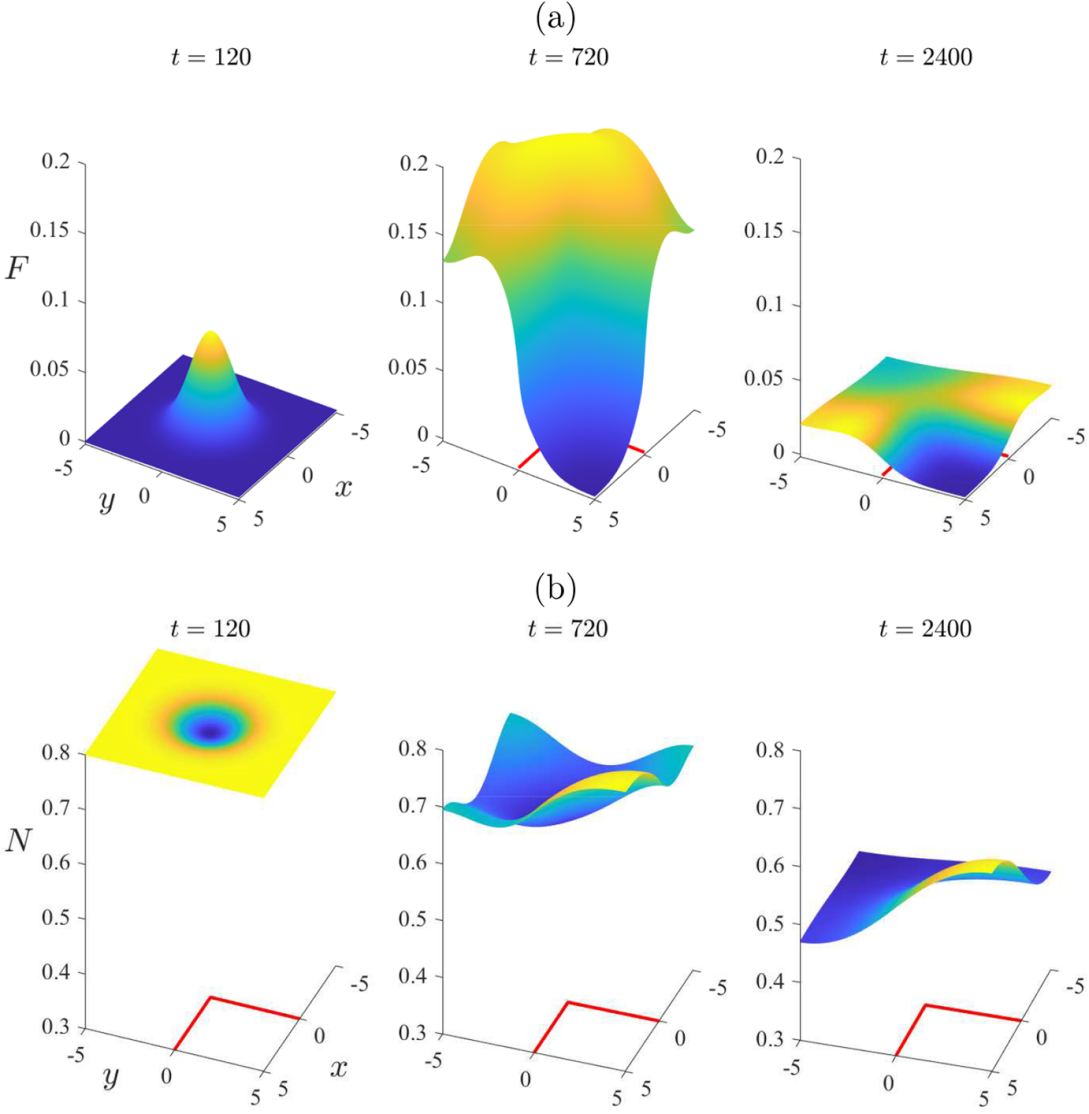
Some spatial patterns for selected times $t=120$, 720 and 2400, for the same (treated) case illustrated in Fig. 10 with multiple treatment periods. There was no ambient treatment ($r_A = 0$) and the treatment rate $r_T = 0.9/7$ was applied over a larger square corner region, indicated with (red) solid lines. This figure illustrates: **a** the mite numbers *F*, and **b** the total wombat numbers *N* (color figure online)

Figure 13 continues this investigation of the effect of the special target region being located at a corner of the environment, with two sides prevented from the effects of wombat migration. Here, the situation is the same as for Fig. 12, except that the size of the corner target region has been increased to the square $0< x < 5$, $0< y < 5$ which now occupies one quarter of the environment region. This square is sketched with solid lines (red online) at the bottom of each diagram. Repeated treatment is given over this region as in Fig. 12, following the schedule outlined in Fig. 9. As previously, there is no ambient treatment, so that $r_A = 0$, and the treatment rate is $r_T = 0.9/7 = 0.1286$ in the targeted region. To ensure high numerical accuracy, $(M,N) = (121,121)$ Fourier coefficients are used in the spectral solution method (45). Sustained reductions in mite numbers, and corresponding increases in total wombat populations, are achievable in the protected target zone; by contrast, when the target zone was centrally located, it was not found possible to maintain the reductions in mite numbers that were achieved at early times, to the same extent.

**Fig. 14:**
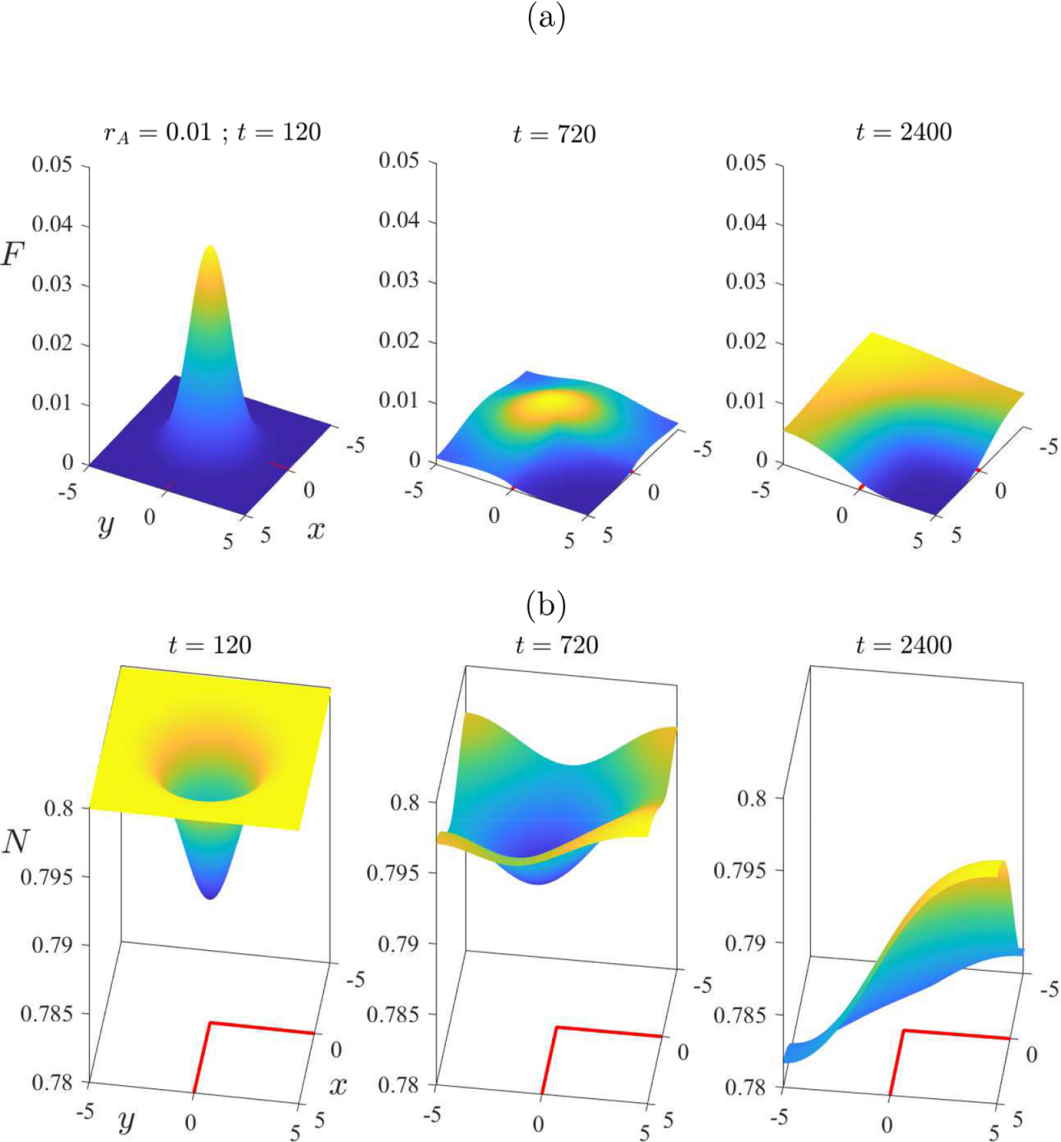
Some spatial patterns for selected times $t=120$, 720 and 2400, with multiple treatment periods applied in the same larger corner region as in Fig. 13. Here, however, there is an ambient treatment rate $r_A = 0.01$ across the entire region, with the treatment rate $r_T = 0.9/7$ from Table 1 used in the target region (indicated with red solid lines). This figure illustrates: **a** the mite numbers *F*, and **b** the total wombat numbers *N* (color figure online)

Finally, we consider the same case as in Fig. 13, except that, now, some ambient treatment over the entire region is supposed to be present, at the low rate $r_A = 0.01$. The results are displayed in Fig. 14. From Figs. 2 and 3 , the “plus” endemic state is still the stable steady state, and this has been confirmed from the linearized solution in Sect. 4. This equilibrium has fomite number density $F^{(+)}_{eq} = 0.0060$ and total wombat number $N^{(+)}_{eq} = 0.7622$, as calculated from (18) and (19). As previously, the calculations are started from the zero-mites steady state (16) for which $N_{eq} = 0.8$, perturbed by the injection of a patch of mites of population size $\varepsilon = 0.2$. The solutions in Fig. 14 were computed with $(M,N) = (121,121)$ Fourier coefficients.

From the last image at time $t = 2400$ in Fig. 14a, it is clear that the primary aim of protecting the wombats in the corner target region (indicated on each diagram) has essentially been accomplished. The mite number density *F* remains very low over that region, over a sustained period of time. Furthermore, from Fig. 14b, the total wombat population *N* continues to remain closer to the mite-free equilibrium value $F_{eq} = 0.8$, rather than falling to the endemic equilibrium value $N^{(+)}_{eq} = 0.7622$. This is a potentially encouraging outcome for wombat survival.

## Discussion

*Sarcoptes scabiei* causes a significant disease burden, on both humanity (World Health Organisation 2020) and a variety of mammalian species across the globe (Astorga et al. 2018). Research addressing management of mange is therefore of value for mitigating this burden. Here, we have sought to explore a bare-nosed wombat population living with endemic mange, and how that population responds to a standard treatment course (Martin et al. 2019) in a restricted treatment area.

The model system we have investigated here predicts three biologically meaningful steady-state configurations. The first is a total extinction state (15), in which the populations of both the mites and the wombats all become zero. This would represent a disastrous outcome for wombat conservation in that area, and must be avoided. Fortunately, we have shown in Sect. 4 that this extinction steady-state possibility is unstable, so that, even if wombat populations become gravely low, the dynamical behaviour of this system will not inevitably drive them to extinction. The second possible steady state is a mite-free configuration. This is surely the most desirable outcome, since the mites are all eliminated and only the wombats remain. An important consideration, then, is to determine under what conditions this mite-free state is stable, which from a practical point of view would imply that if mites were to be introduced, they would eventually all die out. In general this important question can only be answered numerically; however, when the two death rates $\mu _L$ and $\mu _H$ are equal, we have managed to derive, in Theorem 1, a condition which is both necessary and sufficient for the stability of this most desirable steady state. That condition (39) is illustrated graphically in Fig. 3. There is also a third endemic steady state in which both wombats and mites coexist. This is clearly a less desirable outcome, since wombats would suffer the ill effects of mange. (Mathematically, there is also a fourth steady state, but it involves negative populations, and so makes no biological sense).

A linearized approximation to these governing equations was derived in Sect. 4, and the behaviour of its solutions was analyzed. It provides a check on the reliability of computer codes that are devised to solve the governing equations, and it is also used to assess the stability of the equilibrium states. Importantly, there are situations in which small changes to background conditions, as expressed through the parameters in the model, can bring about large-scale qualitative changes to the outcomes for the wombats. In particular, Figs. 2 and 3 indicate that mite-free steady states are possible with appropriate treatment rates, if the treatment can be made to cover the entire environment. By contrast, results such as those in Fig. 11 make clear the fact that, in general, treatment is unlikely to be greatly effective when it only reaches some fraction of the wombat habitat. In practice, it is difficult in the field to know how successful disease management has been, since it is usually difficult to locate all the wombat burrows, or to be certain how efficiently the burrow flaps are working, and so on. At present, there are limited robust field studies that document the management of *S. scabiei* in wombat populations, outside of the study undertaken by Martin et al. (2019).

The linearized solution of Sect. 4 confirmed the veracity of the non-linear results, in circumstances where a population remained reasonably close to an assumed equilibrium state. However, the linearized solution is not capable of predicting a transition from one equilibrium state to another, since it is predicated on the idea that populations consist of a small perturbation to a given equilibrium situation. Under such circumstances, linearization fails after sufficient time has elapsed, since linearized solutions can either only decay or grow exponentially. Nevertheless, they are reasonably accurate even in unstable circumstances at early times, and our linearized solution in Sect. 4 was able to confirm our nonlinear result in unstable situations such as that illustrated in Fig. 6, too, but only at very early times. Later, however, the linearized solution continues to increase exponentially in size, so violating the conditions under which linearization itself is valid. The nonlinear solution, on the other hand, retains its validity at these later times; the solution becomes saturated by nonlinear effects, and at least in some instances, it transitions toward a different equilibrium state.

The behaviour of the nonlinear solution with time can be very difficult to predict, even if it ultimately tends to one of the known equilibrium states in Sect. 3. Its time-dependent behaviour is often sensitively dependent on initial conditions, and this has been illustrated here. The nonlinear solution can undergo large-amplitude excursions, with explosive growth of mite numbers in the shorter term. This was seen in the test case illustrated in Fig. 6, and confirmed with parameters relevant to wombat–mite interactions in Figs. 10, 11, 12 and 13. This could have important ramifications for how biological systems react to sudden changes in their environment, for example. In some circumstances it may be a vital consideration, and is deserving of careful attention in systems such as that studied here; nevertheless, the inherent nonlinearity of the system makes such behaviour very difficult to quantify.

In cases where the naturally-occurring (stable) situation is one in which both wombats and mites co-exist endemically in the environment, even with a low level of ambient treatment against mites, we have investigated situations in which additional higher levels of treatment are applied, but only to certain limited regions in space. The aim, of course, is to reduce mite numbers and boost wombat survivability, at least in those special targeted regions. We have demonstrated, however, that while there is indeed some shorter-term reduction in mite number density in those target regions, the overall spatio-temporal behaviour of the system is somewhat more subtle and nuanced than perhaps expected.

When the targeted treatment region is located centrally within the mite-infested region, the benefits of aggressive treatment within that region, even over repeated time intervals, are rather mixed. Although there is an initial reduction in mite numbers, this becomes considerably less pronounced at later times; furthermore, the increase in overall wombat numbers is almost negligible. However, our calculations have demonstrated that, when it is possible to take advantage of naturally-occurring borders or boundaries within the environment, such as rivers that the wombats are not able to cross, then considerably better outcomes may be possible by locating the targeted treatment region judiciously next to these features. In that case, we have shown that it is possible to achieve almost zero mite numbers within those target regions, and particularly when even a quite low level of ambient treatment is possible throughout the whole region. Furthermore, it is possible to achieve long-term wombat numbers that remain slightly *above* what would be expected from the equilibrium analysis alone (as in Sect. 3), and this was illustrated in Fig. 14. Our model has assumed the wombat habitat to be a perfect rectangle, and although this is highly unlikely to be true in the field, the local reductions in mite numbers we were able to achieve in Fig. 14, near one corner of the region, should be able to be observed in practice. This is because our result is local to a corner and does not rely on changes far away, so that a naturally-occurring similar corner with real impermeable boundaries offers the likelihood of major benefits to wombats in that region.

In this paper, we have modelled wombat (and burrow) distribution as relatively uniform across our simulated spatial environment, so as to limit variables in our model and to support tractable mathematical analysis of the problem. However, wombats are known to have habitat preferences in where they distribute their burrows, and this could be a topic for further research, perhaps even using individual based models. Our field research suggests that wombats generally have fairly fixed and stable home ranges, so our model is likely to represent a reasonable approximation.

There is clearly scope for future work to investigate further strategies to protect wombat populations, based on the location and timing of the treatment regime. Ideally, the entire problem might even be thought of as an optimal control system that is aimed at maximizing wombat numbers, using the treatment function *r*(*x*, *y*, *t*) as the control variable. This would, however, be an enormously ambitious theoretical undertaking that would also make high demands on computer resources, rendering it currently infeasible. Nevertheless, with the careful use of a simulation tool similar to that developed in this paper, combined with detailed knowledge of the terrain and the location of wombats within it provided by dedicated operators in the field, long-term protection of wombat populations from the most severe effects of infestation by sarcoptic mange may be achievable.

## Supplementary information

Highly efficient vectorized code has been developed for the implementation of the nonlinear algorithm presented in Sect. 5 and is presented in Supplementary Material. Further numerical details concerning this code are given in the “Appendix A”.

### Supplementary Information

Below is the link to the electronic supplementary material.

Supplementary file 1 (pdf 98 KB)

## Data Availability

Availability of data and materials

## Appendix A: Vectorized nonlinear algorithm

Evaluation of the spatio-temporal functions *S*(*x*, *y*, *t*), and so on, in the representation (45) at a fixed time *t* is performed in parallel in the program matlab at mesh points $\left( x_p, y_q \right)$, $p = 1, \dots , N_P$, $q = 1, \dots , N_Q$ by creating the doubly-subscripted arrays

A1 $$\begin{aligned} C^X_{m,p} = \cos \left( \frac{m\pi (x_p - L)}{2L} \right) ; \quad C^Y_{n,q} = \cos \left( \frac{n\pi (y_q - B)}{2B} \right) \end{aligned}$$

of sizes $\left( M \times N_P \right)$ and $\left( N \times N_Q \right)$, in which *M* and *N* are the numbers of Fourier coefficients used in the representations (45). With the Fourier coefficients $S_{m,n} (t)$ stored in a matrix $\textbf{S}$, the variable $S \left( x_p,y_q; t \right)$ at each time *t* is obtained in a matrix of size $\left( N_P \times N_Q \right)$ by means of the single matrix operation

$$\begin{aligned} S \left( x_p,y_q; t \right) \equiv \left( \mathbf {C_X} \right) ^T \textbf{S} \mathbf {C_Y}, \end{aligned}$$

and similarly for the other variables in (45).

In addition, each of the expressions in the ordinary differential equations (46)–(48) involves integrals of the form

$$\begin{aligned} J_{k,\ell } (t) = \int _{-B}^{B} \int _{-L}^{L} f(x,y; t) \cos \left( \frac{k\pi (x-L)}{2L} \right) \cos \left( \frac{\ell \pi (y-B)}{2B} \right) \, \textrm{d}x \, \textrm{d}y \end{aligned}$$

at each time *t*. To evaluate this expression, numerical quadrature approximates this in the form

A2 $$\begin{aligned} J_{k,\ell } (t) \equiv \sum _{p=1}^{N_P} \sum _{q=1}^{N_Q} w^X_p w^Y_q f \left( x_p,y_q; t \right) C^X_{k,p} C^Y_{\ell ,q} \end{aligned}$$

in which the expressions defined above in (A1) have been used. Here, the two vectors $w^X_p$ and $w^Y_q$ contain the weight constants for the quadrature rule of choice, and are of sizes $\left( N_P \times 1 \right)$ and $\left( N_Q \times 1 \right)$, respectively.

The double sums in (A2) are likewise evaluated with matrix arithmetic, resulting in a very major reduction in the computing time required. The key to this technique is to define doubly-subscripted elements

A3 $$\begin{aligned} D^X_{m,p} = w^X_p C^X_{m,p}; \quad D^Y_{n,q} = w^Y_q C^Y_{n,q} \end{aligned}$$

of sizes $\left( M \times N_P \right)$ and $\left( N \times N_Q \right)$, respectively, using the expressions in (A1). Now an $( M \times N )$ matrix $\textbf{J}$ can be computed, that contains as its elements the integrals $J_{k,\ell }$ in the Fourier-transformed space with $k = 1, \dots , M$ and $\ell = 1, \dots , N$, using matrix algebra to give

$$\begin{aligned} \textbf{J} = \mathbf {D_X} \textbf{F} \left( \mathbf {D_Y} \right) ^T. \end{aligned}$$

In this expression, the $\left( N_P \times N_Q \right)$ matrix $\textbf{F}$ contains the quantities $f \left( x_p,y_q; t \right)$ at a fixed time *t*. Importantly, the matrices in (A1) and (A3) are stored once, and not recalculated.

## Footnotes

1 Also checked using the computer symbolic-manipulation programme mathematica.

## Code Availability

Sample code is given in the Supplementary Material.
