# Supplementary material for: The effects of spatially-constrained treatment regions upon a model of wombat mange
Source: J Math Biol. 2024 Apr 2;88(5):53. doi: 10.1007/s00285-024-02078-9 (PMC10987376; doi:10.1007/s00285-024-02078-9)
Supplement: Supplementary file 1 — (pdf 98 KB) [file 285_2024_2078_MOESM1_ESM.pdf]

```
%program WombatParallelCornerIntegrator
%This programme calculates nonlinear spatial patterns in the Wombat
%Mange Model.
%We calculate the steady-state populations, and then choose one and
%perturb around it using solutions represented as Fourier series.  We
%solve a system of ODEs for the Fourier coefficients.
%
%The initial conditions will be that everything is at steady state, and
%then we dump a load of mites in a particular area.
%
%Here, I speed up the Algorithm using Matrix Arithmetic,
%as shown to me by Steve Walters.
%
%Here, the treatment rate  r  is not a constant, but is now a function of
% x , y  and  t .   Here, we try to protect Wombats in a corner.
%
%Give the death rates for the wombats.
mu=1/(15*365);
mu_L=1/60;
mu_H=1/60;
%Give the birth rate, susceptible rate and infection rate for wombats.
b=1/(3*365);
beta=0.01;
gamma=1/30;
%Give the growth rate  f  and death rate  mu_F  for the mites.
f=1/5;
mu_F=1/19;
%mu_F=0.005
%Give the relapse rate  k_R .
k_R=1/7;
%
%Give the treatment rate  r  of the wombats, in the ambient and in the
%special region.
rAmbient=0.01
%rAmbient=0
rSpecial=0.9/7
TimeStart=200
TimeFinal=250
%Give the size of the treatment region.
%Give the centre of the rectangular region.
xcentTreat=2.5;
%xcentTreat=4
ycentTreat=2.5;
%ycentTreat=4
%Specify the size of the rectangular treatment region.
xrTreat=2.5;
yrTreat=2.5;
%
%Give the diffusion constant  sigma .
%sigma=1.e-3;
sigma=1/(1.5*365*0.5*0.5)
%
%Calculate the four Equilibrium Points.
%
%State 1A:  total extinction.
SS1A=0;
IL1A=0;
IH1A=0;
RR1A=0;
```

```
FF1A=0;
Ntotal1A=0;
Eq1A=zeros(1,5);
Eq1A(1)=SS1A;
Eq1A(2)=IL1A;
Eq1A(3)=IH1A;
Eq1A(4)=RR1A;
Eq1A(5)=FF1A;
Eq1A
Ntotal1A
%
%State 1B:  only susceptibles remain.
constK=(mu+k_R+rAmbient)/(mu+k_R);
SS1B=(b-mu)/(b*constK);
IL1B=0;
IH1B=0;
RR1B=(b-mu)*rAmbient/(b*(mu+k_R+rAmbient));
FF1B=0;
Ntotal1B=(b-mu)/b;
Eq1B=zeros(1,5);
Eq1B(1)=SS1B;
Eq1B(2)=IL1B;
Eq1B(3)=IH1B;
Eq1B(4)=RR1B;
Eq1B(5)=FF1B;
Eq1B
Ntotal1B
%
%States 2plus and 2minus:  endemic states.
theta_L=mu+mu_L+rAmbient;
theta_H=mu+mu_H+rAmbient;
zeta_H=(f*(theta_H+gamma))/(mu_F*gamma);
constM=(theta_H*(theta_L+gamma))/(gamma*beta*zeta_H);
constN=(gamma*constM*zeta_H)+theta_H+gamma;
constL=(rAmbient*k_R*constN)/(gamma*(mu+k_R))-constM*zeta_H*(beta+mu+rAmbient);
TraceT=b*(constK/gamma)*constN*(1-2*constK*constM)+constL;
DeterD=(b*constK*constN/gamma)^2 ...
    -(4*mu*b*(constK^3)*(constN^2)*constM)/(gamma^2) ...
    +2*constL*b*(constK/gamma)*constN*(1-2*constK*constM)+(constL^2);
coeffIH2=(1/(2*b))*(gamma/(constK*constN))^2;
IH2plus=coeffIH2*(TraceT+sqrt(DeterD));
IL2plus=(theta_H/gamma)*IH2plus;
SS2plus=constM*(1+zeta_H*IH2plus);
FF2plus=zeta_H*IH2plus;
term2plus=gamma*constM*(1+zeta_H*IH2plus)+(theta_H+gamma)*IH2plus;
RR2plus=(rAmbient*term2plus)/(gamma*(mu+k_R));
Ntotal2plus=(constK*term2plus)/gamma;
Eq2plus=zeros(1,5);
Eq2plus(1)=SS2plus;
Eq2plus(2)=IL2plus;
Eq2plus(3)=IH2plus;
Eq2plus(4)=RR2plus;
Eq2plus(5)=FF2plus;
Eq2plus
Ntotal2plus
IH2mins=coeffIH2*(TraceT-sqrt(DeterD));
IL2mins=(theta_H/gamma)*IH2mins;
SS2mins=constM*(1+zeta_H*IH2mins);
FF2mins=zeta_H*IH2mins;
```

```
term2mins=gamma*constM*(1+zeta_H*IH2mins)+(theta_H+gamma)*IH2mins;
RR2mins=(rAmbient*term2mins)/(gamma*(mu+k_R));
Ntotal2mins=(constK*term2mins)/gamma;
Eq2mins=zeros(1,5);
Eq2mins(1)=SS2mins;
Eq2mins(2)=IL2mins;
Eq2mins(3)=IH2mins;
Eq2mins(4)=RR2mins;
Eq2mins(5)=FF2mins;
Eq2mins
Ntotal2mins
%
%
%Now let's choose an equilibrium state, for the INITIAL CONDITION .
SSeq=SS1B;
ILeq=IL1B;
IHeq=IH1B;
RReq=RR1B;
FFeq=FF1B;
Ntotaleq=Ntotal1B;
%SSeq=SS2plus;
%ILeq=IL2plus;
%IHeq=IH2plus;
%RReq=RR2plus;
%FFeq=FF2plus;
%Ntotaleq=Ntotal2plus;
%
%Give the size of our Wombat Reserve.
XL=5;
YB=5;
%Give the size of the region over which mites will be dumped.
%Give the centre of the rectangular region.
xcentre=0;
ycentre=0;
%Specify the size of the rectangular dumping region.
xregion=1;
yregion=1;
%Give the mite density in the dumping region.
EpsMite=0.2;
%
Mmax=121;
Nmax=121;
Mplus1=Mmax+1;
Nplus1=Nmax+1;
M1timesN1=Mplus1*Nplus1;
MNtotalNo=5*M1timesN1;
%
%Give the values of  x .
nxpoints=5*Mmax;
[xx,wtxx]=lgwt(nxpoints,-XL,XL);
%Give the values of  y .
nypoints=5*Nmax;
[yy,wtyy]=lgwt(nypoints,-YB,YB);
%
%Calculate and store  cos(m pi (x-L)/2L) .
cosmpix=zeros(Mplus1,nxpoints);
cosmxwt=zeros(Mplus1,nxpoints);
for im1P=1:Mplus1
    im=im1P-1;
```

```
    for ixx=1:nxpoints
        cosmpix(im1P,ixx)=cos(im*pi*(xx(ixx)-XL)/(2*XL));
        cosmxwt(im1P,ixx)=cosmpix(im1P,ixx)*wtxx(ixx);
    end
end
%
%Calculate and store  cos(n pi (y-B)/2B) .
cosnpiy=zeros(Nplus1,nypoints);
cosnywt=zeros(Nplus1,nypoints);
for in1P=1:Nplus1
    in=in1P-1;
    for iyy=1:nypoints
        cosnpiy(in1P,iyy)=cos(in*pi*(yy(iyy)-YB)/(2*YB));
        cosnywt(in1P,iyy)=cosnpiy(in1P,iyy)*wtyy(iyy);
    end
end
%
deltakLopt=ones(Mplus1,Nplus1);
deltakLopt(1,:)=2;
deltakLopt(:,1)=2;
deltakLopt(1,1)=4;
%
%Give initial conditions for the coefficients of the variables.
%Initially, everything is at its equilibrium value, except for the mites.
SSmncoef=zeros(Mplus1,Nplus1);
EEmncoef=zeros(Mplus1,Nplus1);
HHmncoef=zeros(Mplus1,Nplus1);
RRmncoef=zeros(Mplus1,Nplus1);
FFmncoef=zeros(Mplus1,Nplus1);
SSmncoef(1,1)=SSeq;
EEmncoef(1,1)=ILeq;
HHmncoef(1,1)=IHeq;
RRmncoef(1,1)=RReq;
%Give  Fmn(0,0) .
FFmncoef(1,1)=FFeq+EpsMite*(xregion/XL)*(yregion/YB);
for iL=1:Nmax
    iL1P=iL+1;
    costermY=cos(iL*pi*(YB-ycentre)/(2*YB));

FFmncoef(1,iL1P)=EpsMite*(xregion/XL)*(4/(iL*pi))*costermY*sin(iL*pi*yregion/(2*YB
));
end
for ik=1:Mmax
    ik1P=ik+1;
    costermX=cos(ik*pi*(XL-xcentre)/(2*XL));

FFmncoef(ik1P,1)=EpsMite*(yregion/YB)*(4/(ik*pi))*costermX*sin(ik*pi*xregion/(2*XL
));
end
for ik=1:Mmax
    ik1P=ik+1;
    costermX=cos(ik*pi*(XL-xcentre)/(2*XL));
    sintermX=sin(ik*pi*xregion/(2*XL));
    for iL=1:Nmax
        iL1P=iL+1;
        costermY=cos(iL*pi*(YB-ycentre)/(2*YB));
        sintermY=sin(iL*pi*yregion/(2*YB));
```

```
FFmncoef(ik1P,iL1P)=EpsMite*(16/(ik*iL*(pi^2)))*costermX*sintermX*costermY*sinterm
Y;
    end
end
%
DelkLsquared=ones(Mplus1,Nplus1);
for ik1P=1:Mplus1
    ik=ik1P-1;
    kpion2L=(ik*pi)/(2*XL);
    for iL1P=1:Nplus1
        iL=iL1P-1;
        Lpion2B=(iL*pi)/(2*YB);
        DelkLsquared(ik1P,iL1P)=(kpion2L^2)+(Lpion2B^2);
    end
end
%
%Do Lanczos smoothing on the initial fomite dumped population.
ParamLanczos=0.05;
for ik=1:Mmax
    ik1P=ik+1;
    SmoothK=sin(ik*ParamLanczos)/(ik*ParamLanczos);
    for iL=1:Nmax
        iL1P=iL+1;
        SmoothL=sin(iL*ParamLanczos)/(iL*ParamLanczos);
        FFmncoef(ik1P,iL1P)=FFmncoef(ik1P,iL1P)*SmoothK*SmoothL;
    end
end
%
%
%Pack our coefficients into the vector  vec  of unknowns.
vec=zeros(MNtotalNo,1);
for ik1P=1:Mplus1
    ik=ik1P-1;
    for iL1P=1:Nplus1
        iL=iL1P-1;
        IndexkL=ik*Nplus1+iL1P;
        vec(IndexkL)=SSmncoef(ik1P,iL1P);
        vec(M1timesN1+IndexkL)=EEmncoef(ik1P,iL1P);
        vec(2*M1timesN1+IndexkL)=HHmncoef(ik1P,iL1P);
        vec(3*M1timesN1+IndexkL)=RRmncoef(ik1P,iL1P);
        vec(4*M1timesN1+IndexkL)=FFmncoef(ik1P,iL1P);
    end
end
FF0=vec;
%
SSofXY=zeros(nxpoints,nypoints);
EEofXY=zeros(nxpoints,nypoints);
HHofXY=zeros(nxpoints,nypoints);
RRofXY=zeros(nxpoints,nypoints);
FFofXY=zeros(nxpoints,nypoints);
FFonY0=zeros(nxpoints,1);
NNonY0=zeros(nxpoints,1);
%Integrate the system of ODEs for the Fourier coefficients.
options=odeset('RelTol',1e-5,'AbsTol',1e-7);
tfinal=2400;
nosub=20;
deltspan=tfinal/nosub;
tstart=-deltspan;
```

```
tstop=0;
for isub=1:nosub
    tstart=tstart+deltspan;
    tstop=tstop+deltspan;
    tspan=[tstart tstop];
    [timk,vec]=ode45(@WombatParallelCornerRHS,tspan,FF0,options,xx,wtxx,yy,wtyy,
...
        cosmpix,cosnpiy,deltakLopt,DelkLsquared,cosmxwt,cosnywt, ...
        mu,mu_L,mu_H,b,beta,gamma,f,mu_F,k_R,rAmbient,rSpecial, ...
        sigma,XL,YB,TimeStart,TimeFinal,tfinal,xcentTreat,ycentTreat, ...
        xrTreat,yrTreat,Mmax,Nmax,Mplus1,Nplus1, ...
        M1timesN1,MNtotalNo,nxpoints,nypoints);
    %
    %Re-create the solution.
    ns=size(timk);
    ns1=ns(1);
    %Unpack the vector  vec  of unknowns.
    for ik1P=1:Mplus1
        ik=ik1P-1;
        for iL1P=1:Nplus1
            iL=iL1P-1;
            IndexkL=ik*Nplus1+iL1P;
            SSmncoef(ik1P,iL1P)=vec(ns1,IndexkL);
            EEmncoef(ik1P,iL1P)=vec(ns1,M1timesN1+IndexkL);
            HHmncoef(ik1P,iL1P)=vec(ns1,2*M1timesN1+IndexkL);
            RRmncoef(ik1P,iL1P)=vec(ns1,3*M1timesN1+IndexkL);
            FFmncoef(ik1P,iL1P)=vec(ns1,4*M1timesN1+IndexkL);
        end
    end
    %
    tstop
    %
    %Re-construct the variables.
    SSofXY=cosmpix'*SSmncoef*cosnpiy;
    EEofXY=cosmpix'*EEmncoef*cosnpiy;
    HHofXY=cosmpix'*HHmncoef*cosnpiy;
    RRofXY=cosmpix'*RRmncoef*cosnpiy;
    FFofXY=cosmpix'*FFmncoef*cosnpiy;
    %
    figure(1)
    subplot(1,nosub,isub)
    contour(xx,yy,SSofXY')
    figure(2)
    subplot(1,nosub,isub)
    contour(xx,yy,FFofXY')
    figure(3)
    subplot(1,nosub,isub)
    mesh(xx,yy,FFofXY')
    NtotalofXY=SSofXY+EEofXY+HHofXY+RRofXY;
    figure(4)
    subplot(1,nosub,isub)
    mesh(xx,yy,NtotalofXY')
    %
    %Calculate mites and total wombats on the line  y = 0 .
    for ixx=1:nxpoints
        SumFFcentre=0;
        SumNNcentre=0;
        for im1P=1:Mplus1
            im=im1P-1;
```

```
            for in1P=1:Nplus1
                in=in1P-1;

SumFFcentre=SumFFcentre+FFmncoef(im1P,in1P)*cosmpix(im1P,ixx)*cos(in*pi/2);
SumNNcentre=SumNNcentre+(SSmncoef(im1P,in1P)+EEmncoef(im1P,in1P)+ ...
HHmncoef(im1P,in1P)+RRmncoef(im1P,in1P))*cosmpix(im1P,ixx)*cos(in*pi/2);
            end
        end
        FFonY0(ixx)=SumFFcentre;
        NNonY0(ixx)=SumNNcentre;
    end
    figure(5)
    subplot(1,nosub,isub)
    plot(xx,FFonY0)
    figure(6)
    subplot(1,nosub,isub)
    plot(xx,NNonY0)
    %
    for itotalNo=1:MNtotalNo
        FF0(itotalNo)=vec(ns1,itotalNo);
    end
end
```

```
function Vprime=WombatParallelCornerRHS(timek,vec,xx,wtxx,yy,wtyy, ...
        cosmpix,cosnpiy,deltakLopt,DelkLsquared,cosmxwt,cosnywt, ...
        mu,mu_L,mu_H,b,beta,gamma,f,mu_F,k_R,rAmbient,rSpecial, ...
        sigma,XL,YB,TimeStart,TimeFinal,tfinal,xcentTreat,ycentTreat, ...
        xrTreat,yrTreat,Mmax,Nmax,Mplus1,Nplus1, ...
        M1timesN1,MNtotalNo,nxpoints,nypoints)
%This routine creates the right-hand sides for the ODEs for the Fourier
%coefficients.
%
Vprime=zeros(MNtotalNo,1);
SSmncoef=zeros(Mplus1,Nplus1);
EEmncoef=zeros(Mplus1,Nplus1);
HHmncoef=zeros(Mplus1,Nplus1);
RRmncoef=zeros(Mplus1,Nplus1);
FFmncoef=zeros(Mplus1,Nplus1);
rTreatm=zeros(nxpoints,nypoints);
%
%Unpack the vector  vec  of unknowns.
for ik1P=1:Mplus1
    ik=ik1P-1;
    for iL1P=1:Nplus1
        iL=iL1P-1;
        IndexkL=ik*Nplus1+iL1P;
        SSmncoef(ik1P,iL1P)=vec(IndexkL);
        EEmncoef(ik1P,iL1P)=vec(M1timesN1+IndexkL);
        HHmncoef(ik1P,iL1P)=vec(2*M1timesN1+IndexkL);
        RRmncoef(ik1P,iL1P)=vec(3*M1timesN1+IndexkL);
        FFmncoef(ik1P,iL1P)=vec(4*M1timesN1+IndexkL);
    end
end
%
%Construct the treatment matrix  r = rTreatm .
RSwitch=0;
```

```
TimeTreatStart=TimeStart-TimeFinal;
TimeTreatStop=0;
NumbTreats=floor(tfinal/TimeFinal);
for itreats=1:NumbTreats
    TimeTreatStart=TimeTreatStart+TimeFinal;
    TimeTreatStop=TimeTreatStop+TimeFinal;
    if timek > TimeTreatStart
        if timek < TimeTreatStop
            RSwitch=1;
        end
    end
end
for ixx=1:nxpoints
    for iyy=1:nypoints
        rTreatm(ixx,iyy)=rAmbient;
        if abs(xx(ixx)-xcentTreat) < xrTreat
            if abs(yy(iyy)-ycentTreat) < yrTreat
                rTreatm(ixx,iyy)=rAmbient+RSwitch*(rSpecial-rAmbient);
            end
        end
    end
end
%
%
%Re-construct the variables.
SSofXY=cosmpix'*SSmncoef*cosnpiy;
EEofXY=cosmpix'*EEmncoef*cosnpiy;
HHofXY=cosmpix'*HHmncoef*cosnpiy;
RRofXY=cosmpix'*RRmncoef*cosnpiy;
FFofXY=cosmpix'*FFmncoef*cosnpiy;
%
TotalNN=SSofXY+EEofXY+HHofXY+RRofXY;
betaSFon1F=beta*(SSofXY.*FFofXY)./(FFofXY+1);
TermIntSS=-b*(TotalNN.*(TotalNN-1))-betaSFon1F-rTreatm.*SSofXY;
TermIntEE=betaSFon1F-rTreatm.*EEofXY;
TermIntHH=rTreatm.*HHofXY;
TermIntRR=rTreatm.*(SSofXY+EEofXY+HHofXY);
%
%
%Now create the right-hand sides of the ODEs.
SumIntSS=(cosmxwt*TermIntSS)*cosnywt';
SumIntEE=(cosmxwt*TermIntEE)*cosnywt';
SumIntHH=(cosmxwt*TermIntHH)*cosnywt';
SumIntRR=(cosmxwt*TermIntRR)*cosnywt';
%
for ik1P=1:Mplus1
    ik=ik1P-1;
    for iL1P=1:Nplus1
        IndexkL=ik*Nplus1+iL1P;
        SSExtrakL=-
((sigma*DelkLsquared(ik1P,iL1P))+mu)*SSmncoef(ik1P,iL1P)+k_R*RRmncoef(ik1P,iL1P);
        EEExtrakL=-
((sigma*DelkLsquared(ik1P,iL1P))+mu+gamma+mu_L)*EEmncoef(ik1P,iL1P);
        HHExtrakL=-
((sigma*DelkLsquared(ik1P,iL1P))+mu+mu_H)*HHmncoef(ik1P,iL1P)+gamma*EEmncoef(ik1P,
iL1P);
        RRExtrakL=-((sigma*DelkLsquared(ik1P,iL1P))+mu+k_R)*RRmncoef(ik1P,iL1P);
        FFExtrakL=f*(EEmncoef(ik1P,iL1P)+HHmncoef(ik1P,iL1P))-
mu_F*FFmncoef(ik1P,iL1P);
```

```
Vprime(IndexkL)=(SumIntSS(ik1P,iL1P)/(deltakLopt(ik1P,iL1P)*XL*YB))+SSExtrakL;

Vprime(M1timesN1+IndexkL)=(SumIntEE(ik1P,iL1P)/(deltakLopt(ik1P,iL1P)*XL*YB))+EEEx
trakL;
        Vprime(2*M1timesN1+IndexkL)=-
(SumIntHH(ik1P,iL1P)/(deltakLopt(ik1P,iL1P)*XL*YB))+HHExtrakL;

Vprime(3*M1timesN1+IndexkL)=(SumIntRR(ik1P,iL1P)/(deltakLopt(ik1P,iL1P)*XL*YB))+RR
ExtrakL;
        Vprime(4*M1timesN1+IndexkL)=FFExtrakL;
    end
end
```
